# Genome-Wide Identification, Characterization, and Expression Analysis of Glutamate Receptor-like Gene (*GLR*) Family in Sugarcane

**DOI:** 10.3390/plants11182440

**Published:** 2022-09-19

**Authors:** Jing Zhang, Tianzhen Cui, Yachun Su, Shoujian Zang, Zhennan Zhao, Chang Zhang, Wenhui Zou, Yanling Chen, Yue Cao, Yao Chen, Youxiong Que, Niandong Chen, Jun Luo

**Affiliations:** 1 Key Laboratory of Sugarcane Biology and Genetic Breeding, Ministry of Agriculture and Rural Affairs, National Engineering Research Center for Sugarcane, Fujian Agriculture and Forestry University, Fuzhou 350002, China; 2 Key Laboratory of Genetics, Breeding and Multiple Utilization of Crops, Ministry of Education, National Engineering Research Center for Sugarcane, College of Agriculture, Fujian Agriculture and Forestry University, Fuzhou 350002, China; 3 New Huadu Business School, Minjiang University, Fuzhou 350108, China

**Keywords:** sugarcane, glutamate receptor-like gene (*GLR*), genome-wide analysis, expression pattern, biotic and abiotic stresses

## Abstract

The plant glutamate receptor-like gene (*GLR*) plays a vital role in development, signaling pathways, and in its response to environmental stress. However, the *GLR* gene family has not been comprehensively and systematically studied in sugarcane. In this work, 43 *GLR* genes, including 34 in *Saccharum spontaneum* and 9 in the *Saccharum* hybrid cultivar R570, were identified and characterized, which could be divided into three clades (clade I, II, and III). They had different evolutionary mechanisms, the former was mainly on the WGD/segmental duplication, while the latter mainly on the proximal duplication. Those sugarcane GLR proteins in the same clade had a similar gene structure and motif distribution. For example, 79% of the sugarcane GLR proteins contained all the motifs, which proved the evolutionary stability of the sugarcane *GLR* gene family. The diverse *cis*-acting regulatory elements indicated that the sugarcane *GLRs* may play a role in the growth and development, or under the phytohormonal, biotic, and abiotic stresses. In addition, GO and KEGG analyses predicted their transmembrane transport function. Based on the transcriptome data, the expression of the clade III genes was significantly higher than that of the clade I and clade II. Furthermore, qRT-PCR analysis demonstrated that the expression of the *SsGLRs* was induced by salicylic acid (SA) treatment, methyl jasmonic acid (MeJA) treatment, and abscisic acid (ABA) treatment, suggesting their involvement in the hormone synthesis and signaling pathway. Taken together, the present study should provide useful information on comparative genomics to improve our understanding of the *GLR* genes and facilitate further research on their functions.

## 1. Introduction

As one of the ubiquitous basic amino acids, glutamic acid occupies an important position in protein metabolic processes and is involved in many significant chemical reactions in animals, plants, and microorganisms [1]. As early as the 1950s, it was speculated that glutamate had a neurophysiological role in mammal [2]. It acts as an excitatory neurotransmitter, being regulated by glutamate receptors (*GluRs*) [3]. *GluRs* in animals can be divided into two categories, including ionotropic (*iGluRs*) and metabotropic (*mGluRs*); the former can form cation-selective channels, while the latter can activate biochemical cascades [4,5]. The function of glutamate receptors in animals is mostly concentrated on neuronal communication. However, in 1998, glutamate receptor-like genes (*GLRs*) were first found in *Arabidopsis thaliana*, lacking a nervous system or any structure that allowed neuron-like electrical signaling, where four *AtGLRs* homologous to *iGluRs* were observed [6]. Subsequently, 20 *AtGLRs* were identified in the *A. thaliana* genome [7]. Over the next two decades, *GLRs* were discovered in several other plants, such as 24 *OsGLRs* in *Oryza sativa* [8], 13 *SlGLRs* in *Solanum lycopersicum* [9], 16 *ZmGLRs* in *Zea mays* [10], 36 *GhGLRs* in *Gossypium hirsutum* [11], 32 *MdGLRs* in *Malus domestica* [12], 34 *PbrGLRs* in *Pyrus communis* [13], and 29 *BdGLRs* in *Brachypodium distachyon* [14].

As a model for plant *GLRs*, *AtGLRs* can be divided into three clades through the phylogenetic analysis [15], the same as that for *GLRs* from most plant species, such as *S. lycopersicum* [9], *M. domestica* [12], and *B. distachyon* [14]. When co-constructed with *AtGLRs*, the fourth clade was discovered in some plant species, such as *O. sativa* [8] and *G. hirsutum* [11]. There were also some plant *GLRs* that were classified with less than three clades, such as *Z. mays* [10] which was divided into only two clades. The previous research revealed that AtGLRs had all the characteristic domains encoding the iGluRs, including four transmembrane domains (M1, M2, M3, and M4) and two ligand-binding regions (GlnH1 and GlnH2, i.e., S1 and S2) [6]. Similar protein transmembrane topologies were observed in *O. sativa*, *S. lycopersicum*, and *G. hirsutum* [8,9,11]. M2 is a semi-transmembrane domain with a pore-like structure that was prevalent in other voltage-gated or ligand-gated cation channels, such as K^+^, Na^+^, and Ca^2+^, or cyclic nucleotide cation channels [16]. Interestingly, M2 contained a cationic residue, and most cation channels are usually anionic or polar residues in this region, which indicated that plant GLRs have a unique selection mechanism [12]. Different from the previous construction of the three-dimensional (3D) crystal structure of the ligand-binding domain (LBD) in GLR proteins [16,17,18], the assembly of a complete 3D crystal structure for a full-length AtGLR3.4 sequence was completed for the first time in 2021, which resembled a “Y” structure and could be divided into three layers, namely the upper amino-terminal domain (ATD) layer, the middle LBD layer, and the bottom transmembrane domain (TMD) layer [19].

Plant *GLRs* have evolved a variety of unique physiological functions (Table S1), but without neurophysiological effects [20]. The functional research of *AtGLRs* is the most extensive and can be roughly divided into four categories: firstly, different clades of *GLRs* in *A. thaliana* were overlapped in the expression pattern of organs, including leaf, root, flower, and silique, suggesting the functional overlap [21]; secondly, their roles in the carbon-nitrogen balance [22]; thirdly, abscisic acid (ABA) and abiotic stresses sensing [23]; and last but not the least, the contributions to ionic relations [24]. In addition to the study of *AtGLRs*, *GLRs* in other plants have been shown to be involved in the plant physiological activity. In *O. sativa*, the T-DNA mutant of *OsGLR3.1* caused a phenotype of short-root, and the activity of mutant root meristem was distorted and accompanied with programmed cell death, indicating that *OsGLR3.1* was a regulator of root apex cell proliferation and cell death [25]. In *S. lycopersicum*, *SlGLR3.3* and *SlGLR3.5* can mediate cold domestication-induced cold tolerance by regulating the production of plasma hydrogen peroxide (H_2_O_2_) and redox homeostasis [26]. In *Z. mays*, glutamate signaling can improve the heat tolerance of maize seedlings by *ZmGLRs* channels-mediated Ca^2+^ signaling [27]. In *G. hirsutum*, *GhGLR4.8* was resistance-specific to *Fusarium oxysporum* f. sp. *vasinfectum* race 7 [11].

Sugarcane (*Saccharum* spp.) is one of the most important crops for sugar production, accounting for more than 85% of the total global sugar in China [28]. Cultivating stress-resistant varieties is the most effective and economical way to control environmental stresses [29]. Based on genome-wide databases of the sugarcane wild-type species *Saccharum spontaneum* [30] and *Saccharum* hybrid cultivar R570 [31], stress resistance genes can be excavated and applied in breeding sugarcane cultivars through molecular techniques. *GLRs* have been extensively studied in many species [7,8,9,10,11,12,13] but rarely studied in sugarcane. In the present study, *GLR* genes were identified in *S. spontaneum* [30] and *S.* hybrid R570 [31], and their basic characteristics and evolutionary mechanisms were described and summarized. What is more, the expression of *SsGLRs* in clade III in different sugarcane tissues (bud, leaf, epidermis, stem pith, and root) and in response to phytohormonal (salicylic acid (SA), methyl jasmonic acid (MeJA), and ABA), biotic (*Sporisorium scitamineum*), and abiotic (cold and drought) stresses was determined. This study aims to provide valuable information about the sugarcane *GLR* gene family and set up the basis for its functional characteristics in sugarcane or even other plant *GLRs* in the future.

## 2. Results

### 2.1. Identification and Classification of GLR Gene Family in Sugarcane

In order to identify the sugarcane *GLR* genes, the most typical plant *GLR* gene family in *A. thaliana* (*AtGLRs*) was selected as a template. Four functional domains (IPR001638, IPR044440, IPR001320, and IPR001828) were screened out by InterProScan [32], and the corresponding HMM profiles (PF00497, PF00060, PF01094, and PF10613) were then compared using TBtools (64 bit) [33] (Table 1). In total, 34 SsGLRs and 9 ShGLRs with both these four domains were filtered out from *S. spontaneum* and *S.* hybrid R570, respectively. By building a phylogenetic tree with AtGLRs, these sugarcane GLR protein sequences can be divided into three clades and named according to the chromosome position (Figure S1 and Table S2). Alleles are distinguished by “a”, “b”, and “c” and duplicated genes by “d”, which are respectively termed as the clade I (AtGLR1.1/1.2/1.3/1.4, SsGLR1), clade II (AtGLR2.1/2.2/2.3/2.4/2.5/2.6/2.7/2.8/2.9, SsGLR2.1a/2.1d/2.1b/2.2d/2.3/2.4a/2.4b/2.4c/2.5a/2.5b/2.6/2.7d/2.8/2.9a/2.9b/2.10a/2.10b/2.10c/2.11/2.12/2.13/2.14, Sh2.1/2.2/2.3/2.4/2.5), and clade III (AtGLR3.1/3.2/3.3/3.4/3.5/3.6/3.7/3.8/3.9/3.10/3.11, SsGLR3.1/3.2a/3.2b/3.3/3.4a/3.4b/3.5/3.6a/3.6b/3.7a/3.7b, ShGLR3.1/3.2/3.3/3.4).

### 2.2. Phylogenetic Analysis of GLR Gene Family

**Figure 1:**
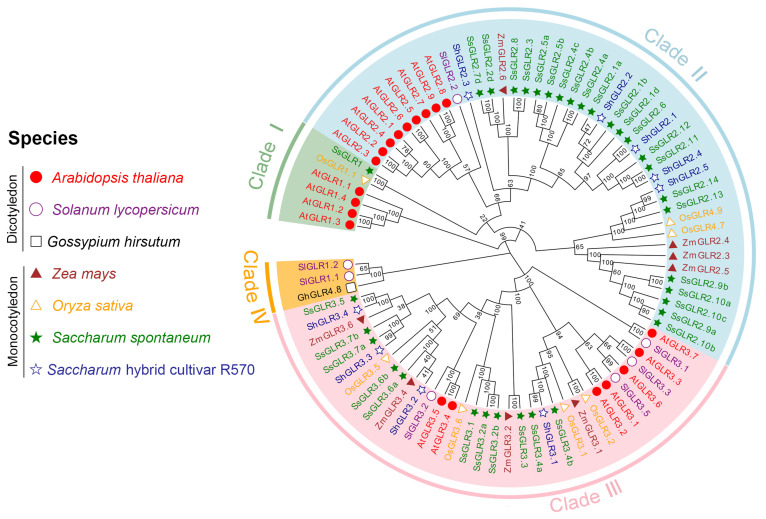
Phylogenetic relationship of GLR proteins in different plant species. The MEGA-X (64 bit) ClustalW with the parameters of gap-opening penalty = 10 and gap-extension penalty = 1 and using the Neighbor-Joining (NJ) method (p-distance model, 1000 ultrafast bootstraps) to conduct the original phylogenetic tree. The number on the branch represented the bootstrap value. All the GLR protein sequences are listed in Table S5.

To study the evolutionary relationship of *GLRs* in sugarcane, a phylogenetic tree was constructed using 86 GLR proteins from three dicotyledons and four monocotyledons (Figure 1 and Tables S2–S5). These GLRs could be divided into four clades (clade I, II, III, and IV). Interestingly, some studies [21] believe that clade I and clade II of AtGLR belong to sister subfamilies, and clade II may have evolved from the gene copy of the clade. In our study, we speculated that the rule may also be applicable to sugarcane GLRs. It is interesting that the GLRs of monocotyledonous and dicotyledonous plants were not clustered separately, indicating that the GLR sequences were conserved among plant species.

### 2.3. Characteristics of GLR Gene Family in Sugarcane

The basic information about the members of the sugarcane *GLR* gene family was analyzed (Table S6). In *S. spontaneum*, the average length of the *SsGLR* nucleotides was about 6.8 kilobase (kb), but the span was between 3 and 18 kb. The minimum amino acids (aa) number of SsGLR protein was 633 (SsGLR2.1b), and the highest was 1388 (SsGLR3.5), mostly from 800 to 1000 aa. The corresponding relative protein molecular weight (MW) of the sugarcane *GLR* gene family ranged from 70.27 (SsGLR2.1b) to 200.49 (SsGLR3.5) kilodalton (kDa). The isoelectric point (*p*I) of the SsGLRs varied from 4.69 (SsGLR3.1) to 8.64 (SsGLR2.5b). The grand average of hydropathicity (GRAVY) analysis showed that hydrophilic and hydrophobic proteins each account for half of the total of SsGLR protein number, which was 17. In *S.* hybrid R570, ShGLR2.1 had the smallest values of nucleotides, coding sequence (CDS), amino acids, and MW, which were 3009 base pair (bp), 1842 bp, 613 aa, and 69.5 kDa, respectively. In addition, the nucleotide length of *ShGLR3.3* was 10385 bp. The CDS, protein numbers, and MW values of ShGLR2.4 were the largest, which were 3603 bp, 1200 aa, and 133.68 kDa, respectively. The *p*I of the ShGLRs ranged from 5.73 (ShGLR2.2) to 9.31 (ShGLR2.1). With respect to GRAVY, three of the ShGLRs (ShGLR2.1/2.2/2.4) were predicted to be hydrophilic proteins, and the remaining were hydrophobic proteins. As with the AtGLRs, subcellular localization predictions suggested that both the SsGLRs and ShGLRs were predominantly located in the plasma membrane.

### 2.4. Secondary Structure of Sugarcane GLR Proteins

The secondary structure of proteins is mainly maintained by hydrogen bonds, which can be divided into alpha helix (Hh), beta turn (Tt), random coil (Cc), and extended strand (Ee) [34]. The one-to-one prediction of the secondary structure of sugarcane GLR proteins was carried out as detailed in Table S7. Whether it was for the *S. spontaneum* or *S.* hybrid R570 GLR proteins, Hh and Cc were the mainstays, accounting for 30 to 45% of the total, respectively. The Tt accounted for the smallest, with an average proportion of 5.43% (Figure S2A). Among the 43 sequences, ShGLR2.4 had a Tt structure accounting for 9.25%, and the rest of the proteins accounted for a rate of 4 to 6.5% (Table S7). Taking SsGLR3.4a as an example (Figure S2B), each amino acid corresponded to a secondary structure, and the sequence had a total of 925 aa. In addition, Hh, Tt, Ee, and Cc accounted for 35.68, 4.97, 20.11, and 39.24% of the total numbers of aa in SsGLR3.4a, respectively.

### 2.5. Tertiary Structure of Sugarcane GLR Proteins

In the present study, all the tertiary structures of the SsGLRs and ShGLRs proteins were predicted based on the 7lzh.1 (SWISS-MODEL template ID) template, which is built on the protein structure of AtGLR3.4 [19]. Except for SsGLR2.7d with the consistency of the 7lzh.1 template as 29.96%, the consistency of the rest of the SsGLR and ShGLR proteins with the 7lzh.1 template were all greater than 30%. These sugarcane GLR proteins with a consistency greater than 30% had a similar protein structure with AtGLR3.4, representing a “Y” structure, which can be divided into three layers (ATD, LBD, and TMD) (Figure S3). Among them, nine proteins (ShGLR2.1 and SsGLR2.1b/2.2/2.4c/2.11/2.13/3.4b/3.5/3.6b) failed to display the membrane position, which was inconsistent with the TMHMM prediction. These proteins with similar structures and “double-loop” membrane localization may have similar functions, and it needed to be verified by experiments.

### 2.6. Transmembrane Domain Analysis of Sugarcane GLR Proteins

**Figure 2:**
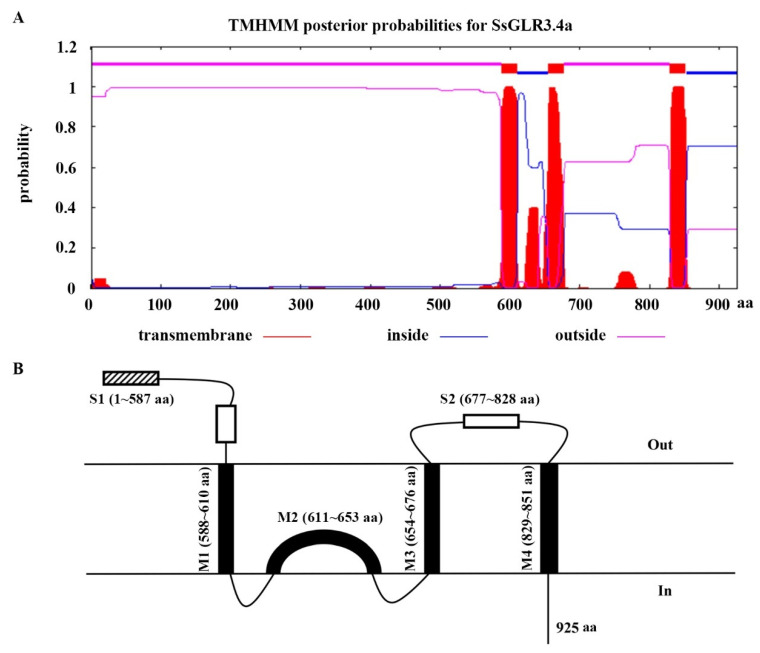
TMHMM transmembrane domain prediction (**A**) and topology structure (**B**), taking SsGLR3.4a as an example. (**A**) Inside represented the intracellular region, and the higher the value, the greater the probability that the amino acid was located in the intracellular region. Outside represented the extracellular region, and the larger the value, the greater the probability that the amino acid was located in the extracellular region. Transmembrane represented the transmembrane region, and the larger the value, the more likely that the amino acid was in the transmembrane region. (**B**) Visualize the topology based on (**A**) with three transmembrane domains (M1, M3, and M4), one M2 domain embedded in the lipid membrane, and two predicted ligand-binding regions S1 and S2 located on the lateral side of the cytoplasm, while “In” represented the intracellular region and “Out” represented the extracellular region.

The TMHMM was used to predict the number of transmembrane domains in each of the GLR proteins of the sugarcane (Table S8 and Figure S4). Among them, 22 SsGLRs and 7 ShGLRs had the similar transmembrane topology domains as *A. thaliana*, including three full transmembrane domains (M1, M3, and M4), one semi-transmembrane domain (M2), and two ligand-binding domains (GlnH1/S1 and GlnH2/S2). Taking SsGLR3.4a as an example (Figure 2), it contained three full transmembrane domains (M1, 588~610 aa; M3, 654~676 aa; and M4,829~851 aa), one domain embedded within the lipid membrane (M2, 611~653 aa), and two predicted ligand-binding regions (S1, 1~587 aa, and S2, 677~828 aa) located laterally in the cytoplasm. Nevertheless, not all sugarcane GLR proteins were found to have similar transmembrane topologies, and no transmembrane domain was observed in the three SsGLRs (SsGLR2.4a/2.11/2.13). Some sugarcane GLR proteins even contained only one transmembrane domain, such as SsGLR2.2 and ShGLR2.1. In *S. spontaneum*, there were even two GLR proteins (SsGLR2.8/2.14) with up to seven transmembrane domains. In *S.* hybrid R570, ShGLR2.5 contained the most domain numbers, which was five.

### 2.7. Conservative Motif and Gene Structure

**Figure 3:**
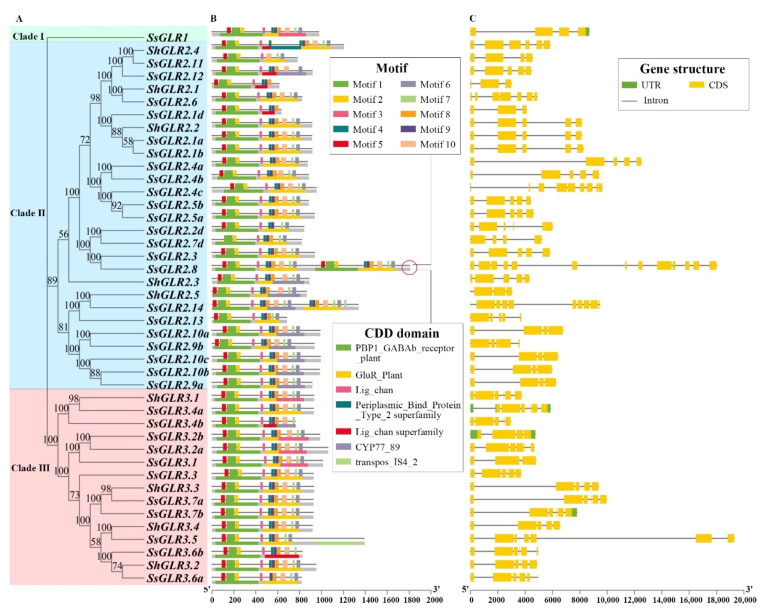
A schematic diagram of phylogenetic tree, conserved motif, and gene structure of SsGLRs and ShGLRs. (**A**) A phylogenetic tree of sugarcane GLRs. The clade name was labeled accordingly and the number on the branch of phylogenetic tree represented the bootstrap value. (**B**) The conservative motif structure of sugarcane GLRs. The 10 motifs were displayed in different colors and correspond one to one in the structural diagram, and the details of each motif were listed in the Table S9. The CDD domain located below motif was searched by Batch-CD. (**C**) The genetic structure of sugarcane *GLRs*. The green box represented the untranslated region (UTR), the yellow box represented the coding sequence (CDS), and the gray line represented the intron (Intron).

The phylogenetic tree of 34 SsGLR and 9 ShGLR proteins were grouped together with conserved motifs (Table S9), CDD domains, and gene structures into one diagram (Figure 3). A total of 10 motifs were identified, of which Motif 1/2/5 contained residues of the ligand-binding domain GlnH1/S1 (PBP1_GABAb_receptor_plant), Motif 4/6/7/9 contained residues of the ligand-binding domain GlnH2/S2 (GluR_Plant/Periplasmic Binding Protein_Type_2_superfamily), and Motif 8/10 represented domain (Lig_chan) residues containing four transmembrane regions (M1, M2, M3, and M4). About 79% of the sugarcane GLR protein sequences contained all motifs, and some members such as SsGLR2.2d/2.11/2.13 lacked the key transmembrane domain Motif 8 or Motif 10, which was consistent with the transmembrane predictions (Figure 3B). As shown in Figure 3C, the intron numbers of the sugarcane *GLRs* were between 2 and 12. In clade I, SsGLR1 contained three introns. The intron numbers of the sugarcane *GLRs* in the clade II ranged from 2 to 12, of which SsGLR2.1 was the least and SsGLR2.8 was the most. The sugarcane GLR of clade III contained four–six introns, of which 71.43% GLRs had five introns. Interestingly, genes with a similar motif distribution also had similar gene structures.

### 2.8. Cis-Acting Regulatory Elements

**Figure 4:**
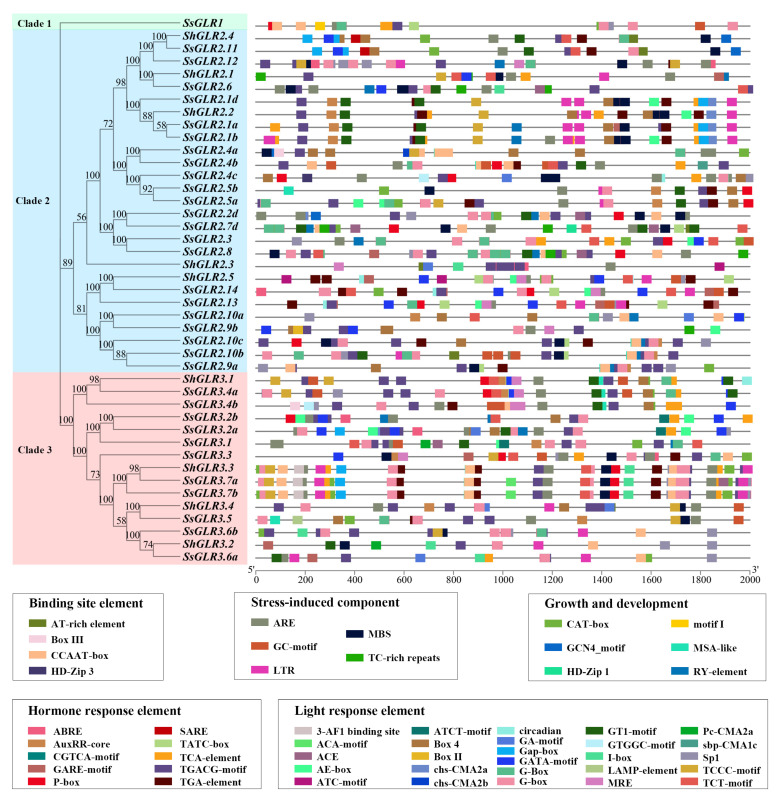
The promoter *cis*-acting regulatory elements in the promoters of *ShGLRs* and *SsGLRs*. Rectangular color blocks with different colors represented different *cis*-acting regulatory elements, which may overlap each other. The number on the branch of phylogenetic tree represented the bootstrap value.

The *cis*-acting regulatory elements of the first 2000 bp upstream fragment of the *ShGLRs* and *SsGLRs* were predicted (Table S10). A total of 100 *cis*-acting regulatory elements were found in the sugarcane *GLR* genes and could be divided into five categories according to their functions. In total, 51 *cis*-acting regulatory elements with 30 different functions were summarized (Figure 4). They were related to the binding site, stress-induced component, growth and development component, hormone response, and light response, each including 4, 5, 6, 10, and 26 *cis*-acting regulatory elements. The CGTCA-motif and TGACG-motif were present in all 42 sugarcane *GLR* genes. Although the sugarcane *GLR* genes involved 51 *cis*-acting regulatory elements, some elements were only found in the *SsGLRs* or *ShGLRs*. For example, the photo-responsive element ATC motif was only found in two *ShGLRs* (*ShGLR2.3*/*2.5*). There were up to nine *cis*-acting regulatory elements found only in *SsGLRs*, including five light-responding elements, the ATCT-motif (*SsGLR3.1*/*3.2a*/*3.2b*), ACA-motif (*SsGLR3.7a*/*3.7b*), Box II (*SsGLR2.3*/*2.5a*/*2.9b*), LAMP-element (*SsGLR3.5*), and sbp-CMA1c (*SsGLR2.4b*); two binding protein-site action elements, Box III (*SsGLR2.4a*/*3.4b*) and HD-Zip 3 (*SsGLR2.4c*); and two growing development elements, HD-Zip 1 (*SsGLR2.7d*) and motif I (*SsGLR1*).

### 2.9. Chromosome Localization, Gene Duplications, and Synteny Analysis

**Figure 5:**
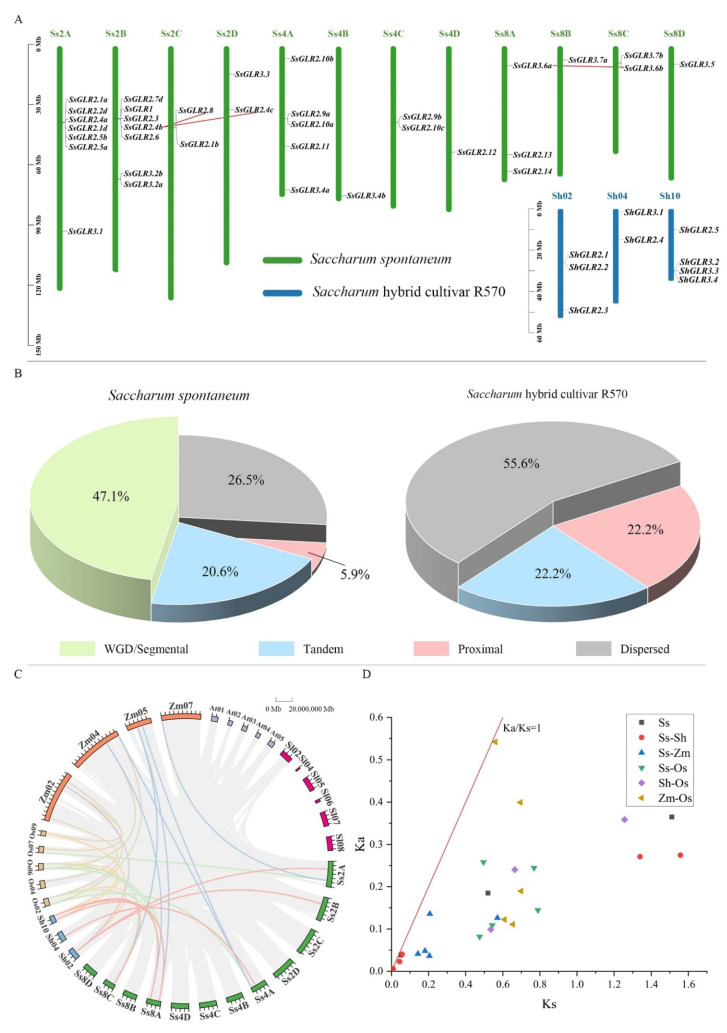
Evolutionary analysis of *SsGLRs* and *ShGLRs*. (**A**) Chromosome localization of *SsGLRs* and *ShGLRs*; each green bar represented chromosome, the left side showed its own chromosome number, and the red line represented the collinear relationship within the species. (**B**) Distribution of gene replication types of *SsGLRs* and *ShGLRs*. WGD represented whole-genome duplications. (**C**) Synteny analysis among six species. The pink line represented homologous *GLR* gene pairs between *S. spontaneum* and *S.* hybrid R570; the green line represented homologous *GLR* gene pairs between *S. spontaneum* and *O. sativa;* the blue line represented homologous *GLR* gene pairs between *S. spontaneum* and *Z. mays*; the orange line represented homologous *GLR* gene pairs between *S.* hybrid R570 and *O. sativa*; the purple line represented homologous *GLR* gene pairs between *S.* hybrid R570 and *Z. mays*; the brown line represented homologous *GLR* gene pairs between *O. sativa* and *Z. mays*. The 0 to 20,000,000 Mb refers to the size of each scale on the chromosome. (**D**) Homologous relationship between species. Y axis and X axis represented synonymous mutation (Ka) and non-synonymous mutation (Ks) values of each pair, and the red line showed Ka/Ks =1; the details of Ka, Ks, and Ka/Ks are listed in Table S12. Chromosome names were named by species abbreviations of *A. thaliana* (At), *S. lycopersicum* (Sl), *O. sativa* (Os), *Z. mays* (Zm), *S. spontaneum* (Ss), and *S.* hybrid R570 (Sh).

To further elucidate the evolutionary relationship of the *GLR* gene family in sugarcane, the chromosomal location, gene duplication, and collinear relationships among species were analyzed (Tables S11 and S12). Figure 5A showed that the *SsGLRs* and *ShGLRs* were unevenly distributed in 12 *S. spontaneum* chromosomes (Ss2A/B/C/D, 4A/B/C/D, and 8A/B/C/D) and 3 *S.* hybrid R570 chromosomes (Sh02/04/10). In *S. spontaneum*, nearly half of the *SsGLR* genes were retained as whole-genome duplications (WGD)/segmental duplication (47.1%), and the rest were distributed in dispersed, proximal, and tandem duplications, accounting for 26.6, 5.9, and 20.6%, respectively (Figure 5B). The *ShGLR* genes were not dominated by the WGD/segmental duplication but by the proximal duplication (55.6%), and half of the remaining genes accounted for the dispersed (22.2%) and tandem (22.2%) duplications (Figure 5B).

There were three pairs of homologous genes (*SsGLR2.4b*/*SsGLR2.8*, *SsGLR2.4b*/*SsGLR2.4c*, and *SsGLR3.6b*/*SsGLR3.6c*) in *S. spontaneum*, but none in *S.* hybrid R570. In addition, six pairs of homologous genes (*SsGLR2.5b*/*ShGLR2.2*, *SsGLR2.3*/*ShGLR2.2*, *SsGLR2.11*/*ShGLR2.4*, *SsGLR2.13*/*ShGLR2.5*, *SsGLR3.6a*/*ShGLR3.2*, and *SsGLR3.6b*/*ShGLR2.2*) between *S. spontaneum* and *S.* hybrid R570 were found (Figure 5C, Table S12). Interestingly, there were two ShGLRs (*ShGLR2.2*/*3.2*) that corresponded to two *SsGLR* homologous genes, suggesting that these genes may play a key role in the evolution of the *GLR* gene family. Furthermore, seven pairs of homologous genes were observed between *S. spontaneum* and *Z. mays*, while only one pair of homologous genes existed in *S.* hybrid R570 (Figure 5C), and there were six and four pairs of homologous genes between *S. spontaneum*/*O. sativa* and *S.* hybrid R570/*O. sativa*, respectively. It can be clearly observed that the *GLRs* did not form a homologous gene pair between the four monocotyledons and two dicotyledons, indicating that the *GLR* homologous gene pairs in the plant were likely to be generated after the differentiation of the dicotyledons and monocotyledonous plants. The ratio of the synonymous mutation to the non-synonymous mutation (Ka/Ks) of the interspecies gene pairs were all less than 1.0, indicating that these *GLR* orthogonal homologous genes may be subjected to a strong purification selection for retention (Figure 5D).

### 2.10. Functional Annotation and Enrichment Analysis of Gene Ontology (GO) and Kyoto Encyclopedia of Genes and Genomes (KEGG) of Sugarcane GLR Genes

**Figure 6:**
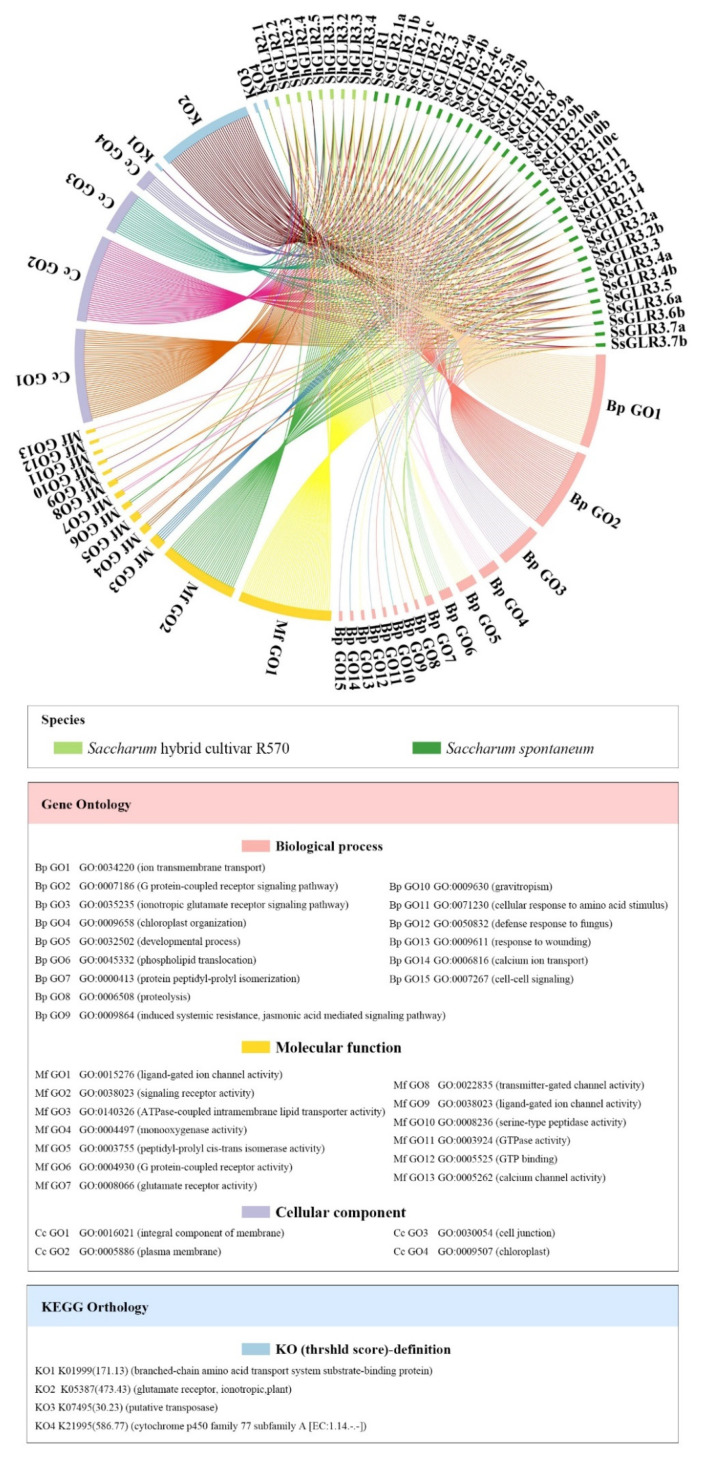
GO and KEGG analyses of GLR proteins from *Saccharum spontaneum* and *Saccharum* hybrid cultivar R570.

The GO annotations of the SsGLR and ShGLR proteins were analyzed (Figure 6 and Table S13). These 43 GLR proteins were involved in 15 biological processes (Bp), 13 molecular functions (Mf), and 4 cell positions (Cc). In the Bp ontology, there were up to 42 proteins involved in the function of ion transmembrane transport (Bp GO1). Among all these sugarcane GLR proteins, only SsGLR3.3 had the function of Bp GO9~15, and SsGLR1 had the function of Bp GO8. In the Mf ontology, in addition to ShGLR2.4, each sugarcane GLR protein was involved in 1~4 Mf functions. The five functions of the Mf ontology were only owned by four sugarcane GLR proteins, including ShGLR2.1 (Mf GO9), SsGLR1 (Mf GO10), SsGLR3.2a (Mf GO11 and Mf GO12), and SsGLR3.3 (Mf GO13). In the Cc ontology, up to 42 GLR proteins were involved in the components of the membrane (Cc GO1) and as few as 8 proteins were involved in chloroplasts (Cc GO4). In summary, the sugarcane GLR proteins may mainly play a role in the transport and transport of substances on the membrane.

For the KEGG enrichment analysis of the SsGLR and ShGLR proteins, only four ontology annotations were found (Figure 6 and Table S14). Among them, K05387, annotated as a plant ionotropic glutamate receptor, was observed in all sugarcane GLR proteins. Meanwhile, for the other three KEGGs (K07495, K01999, and K21995), K07495, annotated as the putative transposase, was found in ShGLR2.4; K01999, annotated as the branched-chain amino acid transport system substrate-binding protein, was found in SsGLR2.8; and K21995, annotated as the cytochrome p450 family 77 subfamily A, was found in SsGLR3.5.

### 2.11. Expression Patterns of SsGLRs in Transcriptome Data

**Figure 7:**
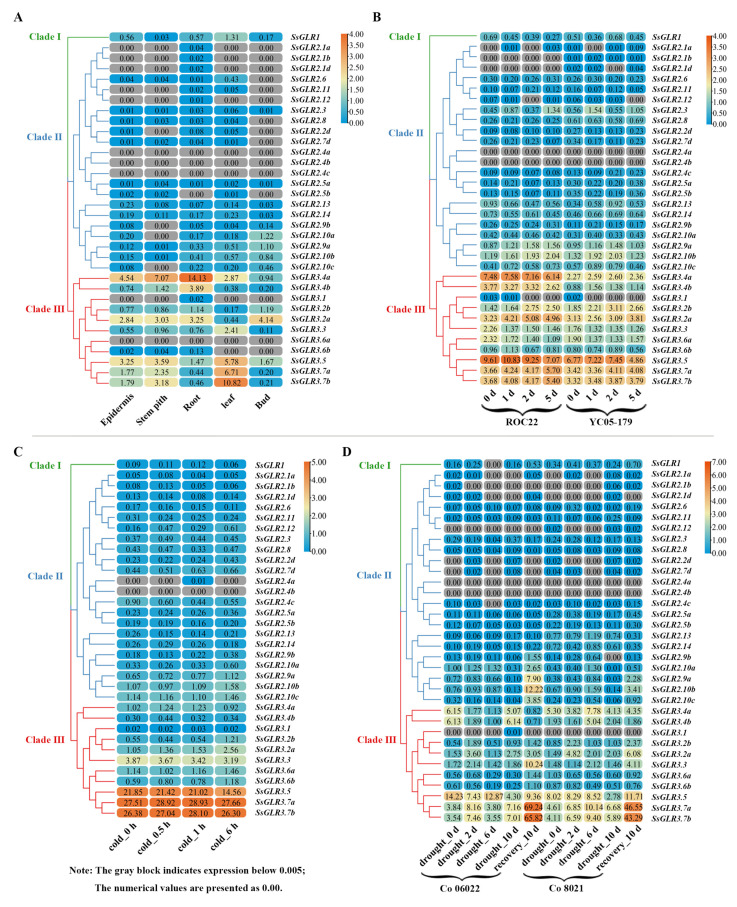
Expression patterns of the sugarcane *GLR* genes from the sugarcane transcriptome data in four different conditions. (**A**) Expression patterns of the *SsGLR* genes in different tissues of ten-month-old sugarcane cultivar ROC22. (**B**) Expression patterns of the *SsGLR* genes in ROC22 (susceptible cultivar) and YC05-179 (resistant cultivar) after smut pathogen infection at 0, 1, 2, and 5 d. (**C**) Expression patterns of the *SsGLR* genes under cold stress. (**D**) Expression patterns of the *SsGLR* genes in Co 06022 (susceptible cultivar) and Co 8021 (resistant cultivar) after 0, 2, 6, and 10 d drought stress and recovery treatment. The color code on the right side of the figure was constructed by TBtools (64 bit), with the transcript level of the *SsGLR* genes transformed as log_2_ FPKM (fragments per kilobase million), ranging from blue (low expression level) to orange (high expression level). The number on the box represented the FPKM value.

Figure 7 demonstrated the gene expression patterns of the sugarcane *GLR* genes based on the sugarcane transcriptome data. As indicated in Figure 7A, the expression of the *SsGLRs* in the different tissues (epidermis, stem pith, root, leaf, and bud) of the sugarcane hybrid cultivar ROC22 at the mature stage was different. The clade I gene (*SsGLR1*) was expressed in all five tissues. The expression of the clade II genes was generally low, and 68% of the *GLR* genes were expressed lowly or unexpressed (fragments per kilobase of transcript per million mapped was less than 0.005, log_2_FPKM < 0.005) in some tissues, of which three genes (*SsGLR2.4a*/*2.4b*/*2.4c*) were unexpressed in all tissues, which was shown as log_2_FPKM < 0.005. The clade III genes had the highest expression abundance among the three clades. In clade III, there were eight genes expressed in five tissues, of which *SsGLR3.4a* showed the highest expression levels in the epidermis, stem, and root than the other genes, so as to the *SsGLR3.7b* in the leaf and *SsGLR3.2a* in the bud.

Figure 7B indicated the expression patterns of the *GLR* genes in ROC22 (susceptible cultivar) and YC05-159 (resistant cultivar) after a smut pathogen infection at 0, 1, 2, and 5 d. A clade I gene (*SsGLR1*) was expressed at different time points in both cultivars. Interestingly, in the ROC22, the expression levels of *SsGLR1* were also increased with the prolongation of the infection time, while those in the YC05-179, at other time points except 2 d were lower than that at 0 d. In clade II, 16 *SsGLR* genes were expressed at all the time points in both cultivars, while two *SsGLR* genes (*SsGLR2.4a*/*2.4b*) were not expressed. The expression trend of these genes was complex. Compared with 0 d, only the expression of *SsGLR2.9a* at other time points of the two cultivars was consistent, and the expression amount was increased. In clade III, only *SsGLR3.1* was expressed at individual time points, and all the other genes were expressed at all the time points of both varieties. Compared with 0 d, the expression of *SsGLR3.2b*/*3.7b* was increased at the two varieties, while the expression of *SsGLR3.3/3.6a* was decreased.

Figure 7C showed the expression patterns of the *SsGLR* genes in the GX87-16 cultivar after cold stress at 0, 0.5, 1, and 6 h. *SsGLR2.4a* was only expressed at 1 h and *SsGLR2.4b* at four time points with lowly or unexpressed levels (log_2_FPKM < 0.005), and the remaining 32 *SsGLR* genes were expressed at all the time points. The expression of two genes (*SsGLR2.9a*/*3.2a*) showed an upward trend with the increase in cold stress time, and three genes (*SsGLR2.6*/*3.3*/*3.5*) showed a downward trend.

In Figure 7D, the expression patterns of the *SsGLR* genes in Co 06022 (susceptible cultivar) and Co 8021 (resistant cultivar) after 0, 2, 6, and 10 d drought stress and recovery treatment were analyzed. A clade I gene (*SsGLR1*) was not expressed (log_2_FPKM < 0.005) at 6 d in Co 06022 under the drought treatment. In clade II, 10 *SsGLR* genes were unregulated at some time points. Among the clade II gene expressed at all the time points, compared with 0 d, the expression levels of *SsGLR2.9a*/*2.10b*/*2.10c* were decreased at 6 d after drought treatment and increased significantly at 10 d after recovery. In clade III, except for *SsGLR3.1*, all the other genes were expressed at all the time points. The expression levels of *SsGLR3.2b*/*3.3*/*3.5*/*3.7a*/*3.7b* were significantly higher than that of other time points at 10 d after recovery. Among them, *SsGLR3.7a*/*3.7b* showed an up-down-up expression pattern under drought stress from 0 d to recovery 10 d.

From above, under these four treatments, the expression patterns of the *SsGLR* genes were complex. Interestingly, the expression levels of the clade III genes were significantly higher than those of the other two clades in the whole, suggesting that the *SsGLR* genes of clade III may play a more critical role in sugarcane growth and in response to biotic and abiotic stresses.

### 2.12. qRT-PCR Analysis of Sugarcane GLR Genes under Hormonal Stresses

**Figure 8:**
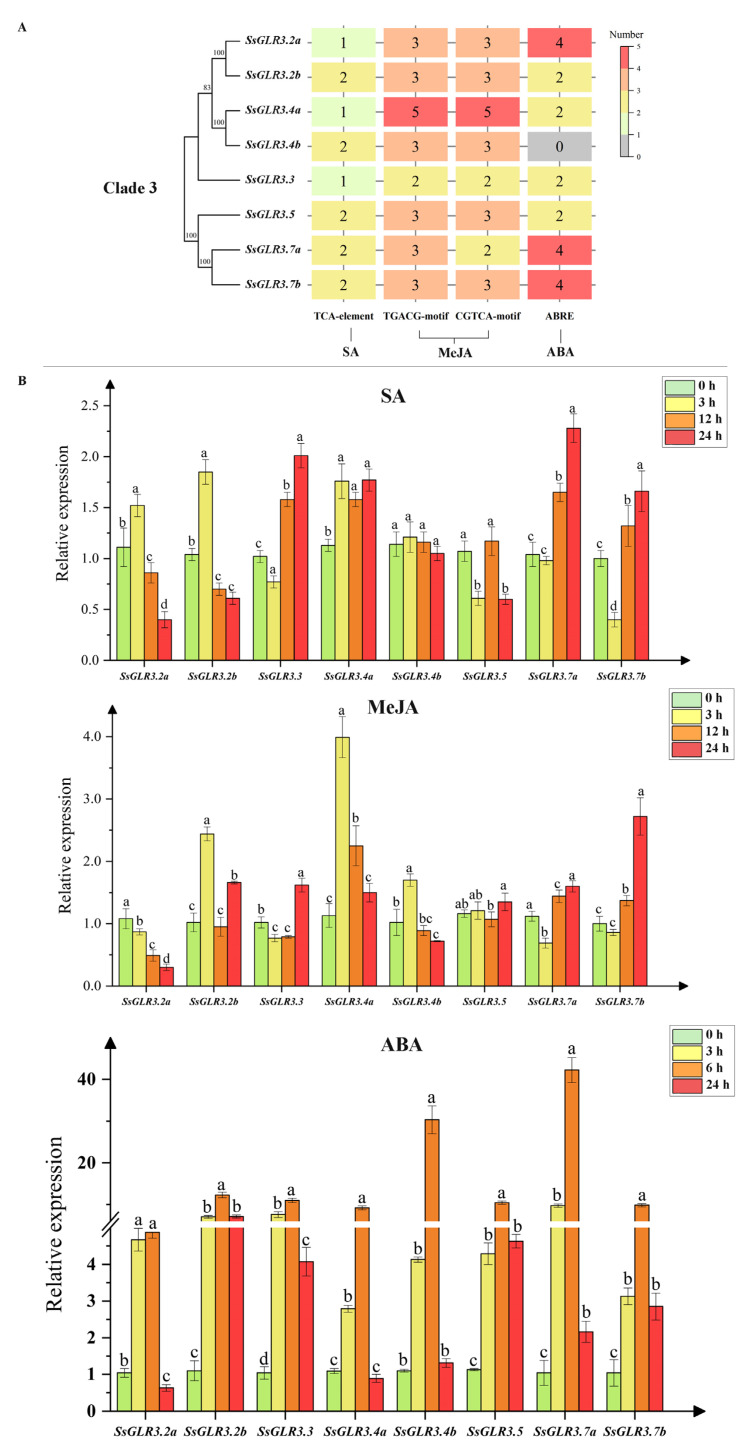
Expression of eight sugarcane *GLR* genes under hormonal stresses revealed by qRT-PCR analysis. (**A**) The *cis*-acting regulatory elements related to hormone response in eight sugarcane *GLR* genes in clade III. The color code on the right side of the figure was constructed by Origin 2021 (OriginLab Corporation, Northampton, MA, USA.) and showed the number of *cis*-acting regulatory elements ranging from gray (low number) to orange (high number). The number on the block represented the number of *cis*-acting regulatory elements. (**B**) The expression levels of eight sugarcane *GLR* genes in clade III under different hormone stresses. Simulated plant hormone stresses conditions, including 5 mM SA, 100 μM MeJA, and 100 μM ABA. All data points were means ± SE (*n* = 3). Different lowercase letters indicated a significant difference, as determined by the least significant difference test (*p*-value < 0.05).

As reported, plant hormones, such as SA, MeJA, and ABA, play an important role in multiple processes of plant growth and development and in response to environmental stress [35,36]. According to the expression patterns of the *SsGLRs* in the transcriptome data, eight sugarcane *GLR* genes in clade III (*SsGLR3.2a*/*3.2b*/*3.3*/*3.4a*/*3.4b*/*3.5*/*3.7a*/*3.7b*) were selected to be tested by quantitative real-time PCR (qRT-PCR) under exogenous hormone stresses (Figure 8). Figure 8A shows the distribution of the hormone-related *cis*-acting regulatory elements in the eight *SsGLRs* of clade III. These elements included the related SA (TCA element), JA (TGACG-motif and CGTCA-motif), and ABA (ABRE) regulatory elements. Except *SsGLR3.4b* which had no ABA-related elements, each gene contained 1~2 SA-related elements, 4~10 MeJA-related elements (2~5 TGACG-motif and 2~5 CGTCA-motif), and 1~5 ABA-related elements, respectively. In Figure 8B, under the SA treatment, there were four groups of genes (*SsGLR3.2a*/*3.2a*/*3.4b, SsGLR3.4a, SsGLR3.5,* and *SsGLR3.3*/*3.7a*/*3.7b*) with similar expression patterns. *SsGLR3.2a*/*3.2b*/*3.4b* expressed the highest at 3 h and then gradually decreased. *SsGLR3.4a* showed an up-down-up expression pattern under SA stress from 0 to 24 h. *SsGLR3.5* showed a down-up-down expression pattern under SA stress from 0 to 24 h. In addition, the expression of *SsGLR3.3*/*3.7a*/*3.7b* decreased at 0–3 h and showed an upward trend at 3–24 h. Under the MeJA treatment, the expression levels of three *SsGLRs* (*SsGLR3.2b*/*3.4a*/*3.4b*) reached a significant peak at 3 h, and *SsGLR3.2a/3.4b* showed a significant downward trend from 3 to 24 h. The expression levels of four *SsGLRs* (*SsGLR3.3*/*3.5*/*3.7a*/*3.7b*) reached a significant peak at 24 h, and the *SsGLR3.7a*/*3.7b* showed a significant upward trend from 3 to 24 h. Under the ABA treatment, eight *SsGLR* genes had similar expression patterns, showing an up-down expression pattern from 0 to 24 h, and peaked at 6 h. Notably, compared with 0 h, *SsGLR3.4a* under the SA treatment and *SsGLR3.2b*/*3.3*/*3.5*/*3.7a*/*3.7b* under the ABA treatment were significantly upregulated at all the time points, while *SsGLR3.2a* under the MeJA treatment was significantly downregulated at all the time points.

## 3. Discussion

*iGluRs* were first discovered in mammal and were intimately linked to the nervous system, with their functions concentrated mainly in neuronal pathways [20]. Plant *GLRs* have evolved plant-specific physiological functions, such as sperm signaling in moss [37], pollen tube growth [38,39], root meristem proliferation [40], and innate immune [41] and wound responses [42]. In this study, a total of 43 *GLR* genes were identified in the *S. spontaneum* and *S.* hybrid R570, and their characteristics were analyzed. Based on transcriptome data and qRT-PCR, the expression patterns of sugarcane *GLR* genes under biotic and abiotic stresses were determined, which should provide a reference for the function identification of *GLRs* in sugarcane.

Proteins with the same domains theoretically have the same biological functions [43]. The law was also followed in screening members of the GLRs. *A. thaliana* was the first plant species used to find *GLRs*. Therefore, the domain of *AtGLR* was served as the reference for searching *GLRs* in almost all plants [8,9,10,13]. The four functional domains (IPR001638, IPR044440, IPR001320, and IPR001828) unique to the AtGLR proteins were filtered by InterProScan [32], and a total of 43 sugarcane *GLRs* in this study also contained these four conservative domains.

An investigation of the origin, evolution, and genetic relationship of species can provide an effective reference for a subsequent genetic operation [8,44,45]. Plant *GLRs* were first discovered in *A. thaliana*, which were divided into three clades [7]. Subsequently, a phylogenetic tree was constructed with iGluRs, bacterial periplasmic-binding proteins (BPBP), which demonstrated that the difference between the iGluRs and AtGLRs was earlier than the iGluRs isoform (NMDA versus AMPA/KA) [7]. In the present study, we divided 86 plant GLRs into four clades (clade I, II, III, and IV) (Figure 1). The SsGLRs were divided into four clades (clade I, II, and III). Interestingly, some studies [21] believe that clade I and clade II of the AtGLR belong to sister subfamilies, and clade II may have evolved from the gene copy of the clade; we speculated that the rule may also be applicable to the SsGLR. ShGLRs were only distributed in three clades (clade II and III), probably because the genomic data for *S.* hybrid R570 are not complete [10]. Three dicotyledonous GLRs (GhGLR4.8 and SlGLR 1.1/1.2) formed a unique new clade, termed clade IV. In addition, the GLRs of monocotyledons and dicotyledons were not clustered separately, indicating that GLR sequences were conserved among plant species. In the collinear analysis, there were also clear differences between dicots and monocots, and no homologous gene pair was formed (Figure 5), so it can be speculated that the *GLRs* in plants most likely occurred after the divergence of dicots and monocots.

The specific spatial structure of a protein affects the biological activity and function of the protein [19]. In our study, the secondary structure of the sugarcane GLRs was mainly dominated by the alpha helix and random coil (Figure S2 and Table S7), which was similar to the results of the SlGLRs [46]. Except for SsGLR2.7d, the tertiary structure of the sugarcane GLRs were almost the same as AtGLR3.4, showing a “Y” structure (Figure S3), suggesting that the sugarcane GLRs and AtGLRs may have similar biological functions. In terms of primary and secondary structures, four transmembrane domains (M1-M4) differed only slightly among the 20 AtGLRs [19]. Across all the clades of the GLRs, the two ligand-binding regions (S1 and S2) were distinctly different [21], with M1, M2, and M3 being the most conserved. It is thus assumed that these conservative domains (M1, M2, and M3) contained important residues that should be helpful to form ion channels and determine the ion selectivity of the channels [47]. Since then, several researchers have concluded that the transmembrane topology of GLRs all have similar structures [6,9]. In this study, 22 SsGLR and 7 ShGLR proteins also had similar transmembrane topology domains to the AtGLRs (Figure 2). This implies that the biological functions of these sugarcane GLRs with similar transmembrane structures may be closer to the AtGLRs. In this study, Motif 8 and Motif 10 represented domain (Lig_chan) residues containing four transmembrane regions (M1, M2, M3, and M4) (Figure 3). Although 79% of the sugarcane GLR proteins contained all 10 motifs, some members, such as SsGLR2.2d/2.11/2.13, lack the key transmembrane domain Motif 8 or Motif 10, which was consistent with the TMHMM prediction (Figure S4).

The SsGLR and ShGLR proteins have similar structures on the same clades, but the evolutionary mechanisms are completely different. Firstly, the replication types for *S. spontaneum* and *S.* hybrid R570 were different; the former was mainly based on the WGD/segmental duplication type, while the latter was mainly based on the proximal duplication (Figure 5B). Tandem duplication plays a major driver, accompanied by more tandem gene microarrays [48]. The expansion of plant *GLRs* in different species is not consistent, and the different types of duplication present in intraspecific genes may also allow subgenus genes to evolve in different directions [13]. Secondly, there were three pairs of homologous *GLR* genes in *S. spontaneum*, but none in *S.* hybrid R570 (Figure 5C). In addition, the *SsGLRs* had more homologous gene pairs between the *ShGLRs* and other species than the *ShGLRs*, perhaps due to the differences in the incomplete genomic data, and similar results were obtained in other studies [44,45,49]. Interestingly, the ratio of Ka/Ks of the homologous *GLR* genes pairs among the six species in this study was less than 1.0 (Figure 7D), which may be the result of a strong purification selection to preserve.

*GLRs* play an indispensable role in the light response, plant growth and development, and response to stresses [47]. Plant hormones are involved in plant growth and development and play an important role in the plant response to environmental stress [35,36]. In our study, the promoters of the sugarcane *GLRs* contained 26 light-responsive elements, 6 growth and developmental-reacting elements, 10 hormone-acting elements, and 5 stress-induced elements (Figure 4 and Table S10). In previous studies, *AtGLR1.1*/*3.5* was found to be involved in the biosynthesis of ABA, thereby controlling the germination process of seeds [23,50]. *RsGluR* played a direct or indirect role in defending against pathogen infection by triggering MeJA biosynthesis [51]. *AtGLR1.2*/*1.3* can actively enhance the cold tolerance of *A. thaliana* by activating endogenous MeJA accumulation and subsequently promoting downstream CBF/DREB1 (a signaling pathway) cold reaction pathways during cold stress [52]. Interestingly, our study found that the promoters of the sugarcane *GLR* genes contained the CGTCA-motif and TGACG-motif elements involved in MeJA regulation, suggesting that *GLRs* may be involved in hormone regulation. Many studies have confirmed that plant *GLRs* play an important role under biotic and abiotic stresses (cold, droughts, etc.) [8,10,11,53]. Liu et al. [11] found that a point mutation in the exon of *GhGLR4.8* was related to the field evolutionary resistance of upland cotton to *Fusarium* wilt, and an RNA-seq analysis showed that knockout of *GhGLR4.8^A^* weakened the cell-wall defense of cotton against *G. hirsutum Fov* race 7. In addition, the expression of *AtGLR3.4* was increased under cold stress [53]. Compared with the control, the expression levels of *Zm2.3*/*3.1* were increased significantly after drought stress [10], and the expression of *OsGLR3.1*/*3.2*/*4.7* was upregulated during panicle development but downregulated under cold, drought, and salt stresses [8]. In our study, one TC-rich repeat involved in the defense and stress response, one LTR element involved in the cryogenic response, and one MBS element involved in drought induction in five stress response elements were found in the promoters of the sugarcane *GLR* genes, indicating that these genes may play a role in biotic and abiotic stresses.

As reported, 20 *AtGLRs* have different degrees of expression in all organs, such as the roots, stems, leaves, flowers, and siliques, and the expression in the roots is the most common [21,54]. Similarly, the expression of the *SsGLRs* was tissue-specific, and up to 85% of the *SsGLR* genes were expressed in the root (Figure 7A). Compared to clade I and clade III, the *AtGLRs* in leaves, flowers, and siliques were concentrated in clade II [21]. In this study, the tissue expression of the *SsGLRs* was mainly observed in the clade III (Figure 7A). When the sugarcane plants with different resistance were infected by the smut pathogen, although the number of copies of the pathogen was different, the quantity of the smut pathogen increased with time [55]. The expression of *SsGLR3.2a*/*3.7b* increased with the increase in infection time of the smut pathogen (Figure 7B), which may signify that these two genes played a positive regulatory role in *S. scitamineum* stress. With the extension of the cold stress time, the expression levels of two genes (*SsGLR2.9a*/*3.2a*) showed an upward trend (Figure 7C), which was similar to the expression pattern of *AtGLR3.4* [53], suggesting that these *GLR* genes played a positive regulatory role in coping with cold stress. In addition, the expression of the three genes (*SsGLR2.6*/*3.3*/*3.5*) showed a downward trend (Figure 7C), which was the same as the expression pattern of *OsGLR3.1*/*3.2*/*4.7*, indicating that these *GLR* genes played a negative regulatory role in response to cold stress. Under drought stress, although the expression levels of the *SsGLRs* in clade III did not show a consistent upward or downward trend with the treatment time, several genes had similar expression patterns. For example, *SsGLR3.7a*/*3.7b* showed an up-down-up expression pattern from 0 d to recovery 10 d, and the expression levels of these two genes increased significantly in recovery 10 d (Figure 7D). Therefore, it is deduced that drought stress may inhibit the expression rate of *SsGLR3.7a/3.7b*. In the transcriptome data, the expression levels of the clade III genes were significantly higher than those of the other two clades, which may explain why the clade III genes had the stronger responsiveness. Previous studies have shown that plant *GLRs* respond to stress by regulating the biosynthesis and signaling of plant hormones [56]. We analyzed the expression patterns of the *SsGLRs* in clade III under plant hormone (SA/MeJA/ABA) stimulation by qRT-PCR (Figure 8B). Compared with 0 h, *SsGLR3.4a* under the SA treatment and *SsGLR3.2b/3.3/3.5/3.7a/3.7b* under the ABA treatment were significantly upregulated at all the time points, and *SsGLR3.2a* under the MeJA treatment was significantly downregulated at all the time points. The above results suggest that these genes were tightly related to hormone biosynthesis or signal transduction.

## 4. Materials and Methods

### 4.1. Identification of Sugarcane GLR Gene Family

Currently, two sugarcane genome databases have been published, which are *S. spontaneum* (http://www.life.illinois.edu/ming/downloads/Spontaneum_genome/) (accessed on 31 July 2021) [30] and *S.* hybrid R570 (http://sugarcane-genome.cirad.fr/) (accessed on 31 July 2021) [31]. In addition, the 20 published *AtGLRs* (TAIR: https://www.arabidopsis.org) (accessed on 31 July 2021) and a number of plant *GLRs* that have been validated for function were selected from *S. lycopersicum* (*SlGLRs*, Sol Genomics Network: https://solgenomics.net/) (accessed on 26 August 2021), *O. sativa* (*OsGLRs*, Rice Genome Annotation Project: http://rice.uga.edu/index.shtml) (accessed on 22 August 2021), *Z. mays* (*ZmGLRs*, MaizeGDB: https://maizegdb.org/) (accessed on 22 August 2021), and *G. hirsutum* (*GhGLRs*, NCBI: https://www.ncbi.nlm.nih.gov/) (accessed on 25 August 2021) (Table S1, Table S3–S5). Take the well-characterized plant *AtGLR* gene family as a reference, *SsGLRs* and *ShGLRs* were identified from *S. spontaneum* and *S.* hybrid R570, respectively. The protein sequences of *AtGLRs* were analyzed by InterProScan [32] (http://www.ebi.ac.uk/interpro/) (accessed on 5 August 2021), and four common conservative domains (IPR001638, IPR044440, IPR001320, IPR001828) were screened out. The Hidden Markov Model (HMM) profile was downloaded from the PFAM database [57] (http://pfam.sanger.ac.uk/) (accessed on 05 August 2021). TBtools (64 bit) [33] was used to blast the HMM profile with the whole genome of sugarcane, and the putative *GLR* protein sequences were submitted to InterProScan [32] and SMART [58] (http://smart.embl-heidelberg.de) (accessed on 06 August 2021) to confirm the domain. The too short sugarcane *GLRs* with nucleic acid sequence smaller than the CDS sequence were eliminated to obtain the final candidate *GLR* genes for the next step of analysis.

### 4.2. Multiple Sequence Alignment and Phylogenetic Analyses

A total of 86 GLR protein sequences were aligned using MEGA-X (64 bit) [59] ClustalW with the following parameters: gap opening penalty = 10 and gap extension penalty = 1, using the Neighbor-Joining (NJ) method (p-distance model, 1000 ultrafast bootstraps) to construct the phylogenetic tree. EvolView [60] (https://www.evolgenius.info/evolview/#login) (accessed on 4 September 2022) was used to visualize and beautify the phylogenetic tree.

### 4.3. Analysis of the Main Characteristics of GLR Gene Family

The biophysical properties, including the MW, GRAVY, *p*I, and instability index of the GLR proteins, were anticipated by the ExPASy database [61] (http://web.expasy.org/protparam/) (accessed on 10 September 2021). Subcellular localizations and protein secondary structure of the GLR proteins were predicted by ProtComp9.0 [62] (http://www.softberry.com) (accessed on 10 September 2021) and SOPAM [34] (http://npsa-pbil.ibcp.fr/cgi-bin/npsa_automat.pl?page=npsa_sopma.html) (accessed on 10 September 2021), respectively. In addition, the protein tertiary structure was predicted by SWISS-Model [63] (https://swissmodel.expasy.org/) (accessed on 10 September 2021), with the consistency of template sequence (the SWISS-MODEL Template Library is searched in parallel with both BLAST and HHblits to identify templates and to obtain target–template alignments) to the target sequence greater than 30%, and the model with closet GMQE value to 1. Transmembrane regions were predicted by TMHMM version 2.0 [64] (http://www.cbs.dtu.dk/services/TMHMM/) (accessed on 31 July 2021), and topology structures were drawn using Powerpoint.

### 4.4. Conserved Motif and Gene Structure Analysis

MEGA-X (64 bit) [59] was used to construct a phylogenetic tree of SsGLRs and ShGLRs. Search for the conserved motif of protein sequences through the online website MEME [65] (http://meme-suite.org/tools/meme) (accessed on 4 September 2021), set the number of motifs to 10, the optimal width from 6 to 100, and the rest of the parameters were default. The gene structure showed the CDD domain and the internal and external exons, by searching and downloading files in the CDD database [66] (https://www.ncbi.nlm.nih.gov/cdd) (accessed on 5 September 2021). Information about the internal and external exon structures was obtained from the gff3 annotation file and visualized with TBtools (64 bit) [33].

### 4.5. Cis-Acting Regulatory Elements Analysis

Based on the *S. spontaneum* [30] and *S.* hybrid R570 [31] genome databases, the 2000 bp of genomic DNA sequence upstream of the translation start site of *SsGLRs* and *ShGLRs* was treated as the promoter sequence, and the *cis*-acting regulatory elements in the promoter regions were identified by the PlantCARE online software [67] (http://bioinformatics.psb.ugent.be/webtools/plantcare/html/) (accessed on 4 December 2021). After classification, it was displayed with TBtools (64 bit) and Origin 2021 (OriginLab Corporation, Northampton, MA, USA.).

### 4.6. Chromosome Localization, Gene Duplications, and Synteny Analyses

The gff3 annotation file of autopolyploid *S. spontaneum* [30] and the monoploid *S.* hybrid R570 [31] were used for the chromosomal localization of sugarcane GLRs and were displayed in TBtools (64 bit) [33]. Detection and classification of duplicate genes and collinear blocks were conducted by using the Multiplex Collinear Scanning Toolkit (MCScanX) with default parameters [68]. Simple Ka/Ks calculator (NG) in TBtools (64 bit) [33] was used to calculate the ratio between homologous gene pairs, and Origin 2021 (OriginLab Corporation, Northampton, MA, USA.) was applied to show the ratio of Ka/Ks between species. Based on the gff3 annotation file of each species, the single-species replication was provided and demonstrated by collinear blocks, and the collinear analysis between genomes was constructed using “Advanced Circo” in TBtools (64 bit) [33].

### 4.7. GO and KEGG Orthology Analysis

GO analysis of GLR proteins was conducted by using PANNZER [69] (http://ekhidna2.biocenter.helsinki.fi/sanspanz/) (accessed on 6 March 2022), and KEGG orthology analysis was carried out through using KofamKOALA [70] (https://www.genome.jp/tools/kofamkoala/) (accessed on 6 March 2022). The data were shown in the “Advanced Circo” in TBtools (64 bit) [33].

### 4.8. Transcriptome Data Analysis

The transcriptome data of different sugarcane tissues, including bud, leaf, epidermis, stem pith, and root from ten-month-old sugarcane cultivar ROC22, were derived from the transcriptome of our group (unpublished). The transcriptome data of different sugarcane cultivars under the stress of *S. scitamineum* were obtained from Que et al. [71], and the materials were selected from ROC22 (susceptible cultivar) and Yacheng 05-159 (YC05-159, resistant cultivar) with the bud samples infected by *S. scitamineum* for 0, 1, 2, and 5 d. The cold stress transcriptome data (PRJNA636260) were obtained from Huang et al. [72], and the selected material was GX87-16 cultivar, which was treated at low temperature two weeks after pot planting, and the leaf samples of 0, 0.5, 1, and 6 h after treatment were taken. The drought stress transcriptome data (PRJNA590595) of Co 06022 (susceptible cultivar) and Co 8021 (resistant cultivar) were obtained from Selvi et al. [73]. The water shortage treatment was carried out after 60 days of pot planting, and the leaf samples of 2, 6, 10, and 10 d of rehydration were taken, respectively. Data collation methods can refer to Cen et al. [44], during which the transcriptome original data were converted into the expression of the *SsGLRs* by log_2_ FPKM (fragments per kilobase of transcript per million mapped) and were rounded to retain two digits after the decimal point of the value. Heat maps were constructed with TBtools (64 bit) [33], with gray patches representing values less than 0.005, and data were presented at 0.00.

### 4.9. qRT-PCR Analysis

Based on the transcriptome data, eight sugarcane *GLR* genes in clade III were selected. Their expression levels in ROC22 under the exogenous hormone stresses of SA, MeJA, and ABA were analyzed by qRT-PCR. The treatment of plant materials refers to the method of Huang et al. [74] with minor changes. The four-month-old ROC22 plantlets were cultured in water for a week, and then treated by exogenous treatment by soaking roots at 28 °C for 16 h in light and 8 h in darkness. Simulated plant hormone stresses conditions include 5 mM SA, 100 μM MeJA, and 100 μM ABA. The leaves were sampled at 0 h, 3 h, 12 h, and 24 h after SA and MeJA treatment, and at 0 h, 3 h, 6 h, and 24 h after ABA treatment [75,76]. Each treatment was biologically repeated three times and stored at −80 °C for total RNA extraction. The methods of total RNA extraction, first strand cDNA synthesis, and qRT-PCR analysis were consistent with the research of Su et al. [77]. According to report of Ling et al. [78], glyceraldehyde-3-phosphate dehydrogenase (*GAPDH*) was found to be the most stable gene among the 13 candidate reference genes in different sugarcane tissues and under various adversity stresses. Therefore, this *GAPDH* housekeeping gene was selected as the only internal reference [78,79]. The 2^−ΔΔCt^ method was used to analyze the relative expression levels of qRT-PCR data [80], and IBM SPSS Statistics 26 software (International Business Machines Corporation, Armonk, NY, USA.) was used for statistical analysis. The data were expressed as mean ± standard error (SE). Significance (*p*-value < 0.05) was calculated using one-way ANOVA followed by Duncan’s new multiple range test. The primer information is listed in Table S15.

## 5. Conclusions

In the present study, a genome-wide bioinformatics analysis of the *GLRs* in sugarcane was carried out systematically for the first time. In total, 43 *GLR* genes, including 34 in *S. spontaneum* and 9 in *S.* hybrid R570, were identified and characterized, which could be divided into three clades (clade I, II, and III). They had different evolutionary mechanisms; the former was mainly on the WGD/segmental duplication, while the latter was mainly on the proximal duplication. The secondary structure, tertiary structure, and motif structure of most of the sugarcane GLR proteins have similar compositions. In addition, the GO and KEGG analyses further predicted the transmembrane transport function of the sugarcane *GLR* gene family. The *cis*-acting regulatory element analysis showed that there were many elements related to binding site, stress-induced, growth and development, hormone response, and light response, which suggested that the *GLR* genes might participate in several types of regulation and transcription. The transcriptome data indicated that the clade III genes had higher expression than the clade I and clade II genes. Furthermore, the qRT-PCR analysis demonstrated that the expression levels of *SsGLR3.4a* under the SA treatment and *SsGLR3.2b*/*3.3*/*3.5*/*3.7a*/*3.7b* under the ABA treatment at other time points were significantly higher than those at 0 d, while the expression of *SsGLR3.2a* under the MeJA treatment was significantly downregulated at all time points, suggesting that these genes were involved in hormone synthesis and signal conversion. To sum up, the present study provides a new insight into the evolution and expression of the sugarcane *GLR* gene family, which should set up a theoretical basis for the further molecular cloning and functional verification of these *GLR* genes in sugarcane.

## Figures and Tables

**Table 1:** Prediction of domains and numbers of GLRs in *Arabidopsis thaliana* and sugarcane.

Type	ID	Description	AtGLR	SsGLR	ShGLR
InterPro	IPR001638	Extracellular solute-binding protein, family3	20	34	9
	IPR044440	Periplasmic ligand-binding domain	20	34	9
	IPR001320	Ionotropic glutamate receptor	20	34	9
	IPR001828	Extracellular ligand-binding receptor	20	34	9
	IPR019594	Ionotropic glutamate receptor, L-glutamate, and glycine-binding domain	1	0	0
	IPR002559	Transposase, IS4-like	0	0	1
CDD	cd19990	PBP1_GABAb_receptor_plant	20	34	9
PFAM	PF00060	Lig_chan	19	33	8
	PF00497	SBP_bac_3	19	34	9
	PF01094	ANF_receptor	20	34	9
	PF10613	Lig_chan-Glu_bd	1	0	0
	PF01609	DDE_Tnp_1	0	0	1

The ID represented the domain name in InterPro database and Conserved Domain Database (CDD) and as the HMM file name in PFAM database. The last three columns of AtGLR, SsGLR, and ShGLR represented the number of GLR proteins in these domains or HMM files in Arabidopsis thaliana, Saccharum spontaneum, and Saccharum hybrid cultivar R570, respectively.

## Supplementary Materials

The following supporting information can be downloaded at: https://www.mdpi.com/article/10.3390/plants11182440/s1, Figure S1: Phylogenetic relationship of GLR proteins in *Arabidopsis thaliana* (At), *Saccharum spontaneum* (Ss), and *Saccharum* hybrid cultivar R570 (Sh), Figure S2: Prediction of protein secondary structure of sugarcane GLR proteins, Figure S3: Prediction of tertiary structure, Figure S4: The transmembrane domains of SsGLRs and ShGLRs predicted by TMHMM, Table S1: Functions of the purported GLR proteins involved in phylogenetic tree, Table S2: The homology matrix of 43 sequences in *S.* hybrid R570 and *S. spontaneum,* Table S3: Species source and protein sequences accession numbers used in this study, Table S4: Renaming of ZmGLRs in this paper, Table S5: The protein and nucleotide sequences used in this study, Table S6: The detailed information of sequence physicochemical properties of *GLR* genes in *S.* hybrid R570 and *S. spontaneum*, Table S7: Secondary structure of GLR proteins in *S.* hybrid R570 and *S. spontaneum*, Table S8: The transmembrane prediction of GLR proteins in *A. thaliana*, *S.* hybrid R570, and *S. spontaneum*, Table S9: The detailed information of motifs in GLR proteins in *S.* hybrid R570 and *S. spontaneum*, Table S10: Promoter *cis*-acting regulatory element analysis of the *GLR* genes in *S.* hybrid R570 and *S. spontaneum*, Table S11: Type of *GLR* genes replication in *S.* hybrid R570 and *S. spontaneum*, Table S12: Collinearity relationship among *GLR* genes in different species, Table S13: The GO enrichment analysis of *GLR* genes in *S.* hybrid R570 and *S. spontaneum*, Table S14: The KEGG enrichment analysis of *GLR* genes in *S.* hybrid R570 and *S. spontaneum*, and Table S15: The primers used in this study [81,82,83,84,85,86,87,88,89].
