# Supplementary material for: Genome-Wide Identification, Characterization, and Expression Analysis of Glutamate Receptor-like Gene (GLR) Family in Sugarcane
Source: Plants (Basel). 2022 Sep 19;11(18):2440. doi: 10.3390/plants11182440 (PMC9506223; doi:10.3390/plants11182440)
Supplement: Supplementary file 1 [file plants-11-02440-s001.zip › Supplementale Figure S1-S4.pdf]

Genome-Wide Identification, Characterization and Expression Analysis of Glutamate Receptor-Like Gene (*GLR*) Family in Sugarcane

Jing Zhang [1,2], Tianzhen Cui [1,2], Yachun Su [1,2], Shoujian Zang [1,2], Zhennan Zhao [1,2], Chang Zhang [1,2], Wenhui Zou [1,2], Yanling Chen [1,2], Yue Cao [1,2], Yao Chen [1,2], Youxiong Que [1,2], Niandong Chen [3,*] and Jun Luo [1,2,*]

1 Key Laboratory of Sugarcane Biology and Genetic Breeding, Ministry of Agriculture and Rural Affairs, Fujian Agriculture and Forestry University, Fuzhou, Fujian, China; zhashoveljing@163.com (J.Z.); ctz5116@126.com (T.Z); sys2009mail@163.com (Y.S.); zangshoujian2020@163.com (S.Z.); Zhangchang@163.com (C.Z.); zwh19961546644@126.com (W.Z.); zn2008@139.com (Z.Z.); caoyue36066@163.com (Y.C.); chenyanling1218@163.com (Y.L.);chen2929yao@163.com (Y.C.); queyouxiong@fafu.edu.cn (Y.Q.)

2 Key Laboratory of Genetics, Breeding and Multiple Utilization of Crops, Ministry of Education, College of Agriculture, Fujian Agriculture and Forestry University, Fuzhou, Fujian, China;

3 New Huadu Business School, Minjiang University, Fuzhou 350108, Fujian, China

* Correspondence: 171944938@qq.com (N.C.); sisluo@126.com (J.L.); Tel.: +86-591-8385-2547 (N.C. & J.L.)

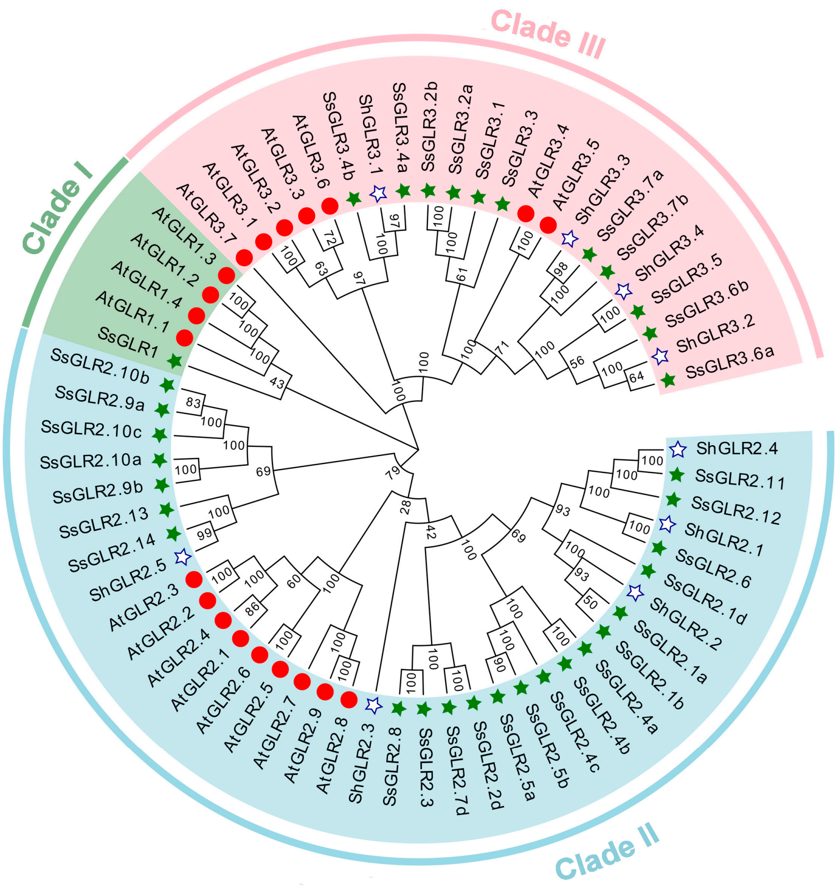

Figure S1. Phylogenetic relationship of GLR proteins in *Arabidopsis thaliana* (At), *Saccharum spontaneum* (Ss) and *Saccharum* hybrid cultivar R570 (Sh). The MEGA-X (64bit) ClustalW with the defalut parameters (gap opening penalty = 10 and gap extension penalty = 1), and using the Neighbour-Joining (NJ) method (p-distance model, 1000 ultrafast bootstraps) to conduct the original phylogenetic tree. The number on the branch represented the bootstrap value. All the GLR protein sequences were listed in Table S5.

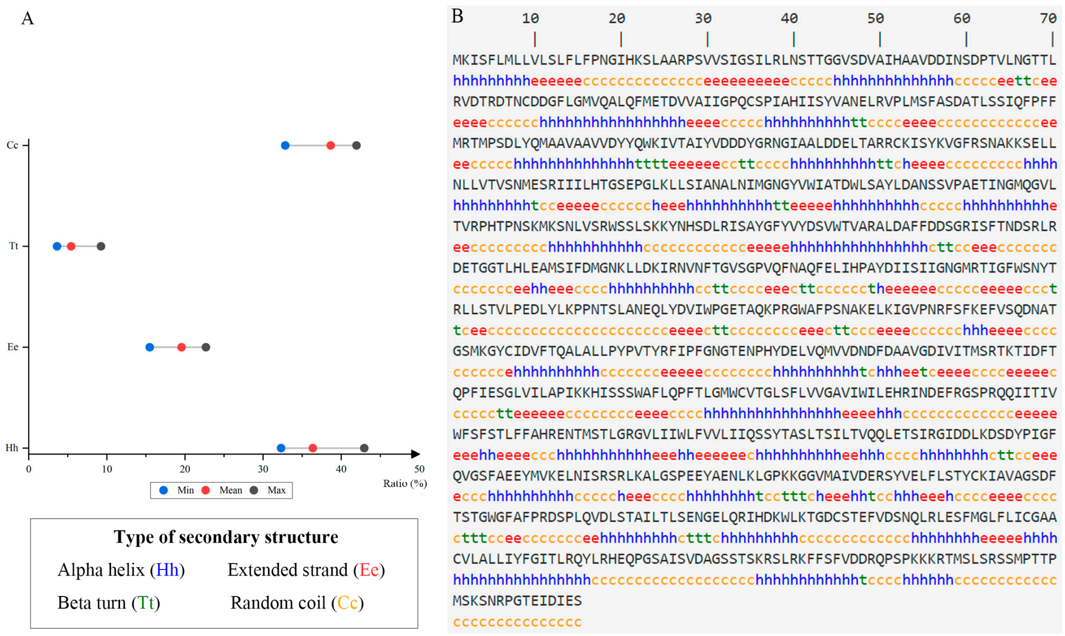

Figure S2. Prediction of protein secondary structure of sugarcane GLR proteins. (**A)** Distribution ratio of protein secondary structure types in sugarcane GLRs. The ordinate represented the secondary structure type, and the abscissa represented the proportion of each secondary structure type in sugarcane GLRs, including the minimum value (min), mean value (mean), and maximum value (max). **(B)** With SsGLR3.4a as an example, the structure prediction diagram of one-to-one correspondence between amino acid sequence and secondary structure. The first row of black capital letters were amino acids, and the second row of colored small letters were the corresponding secondary protein structure.

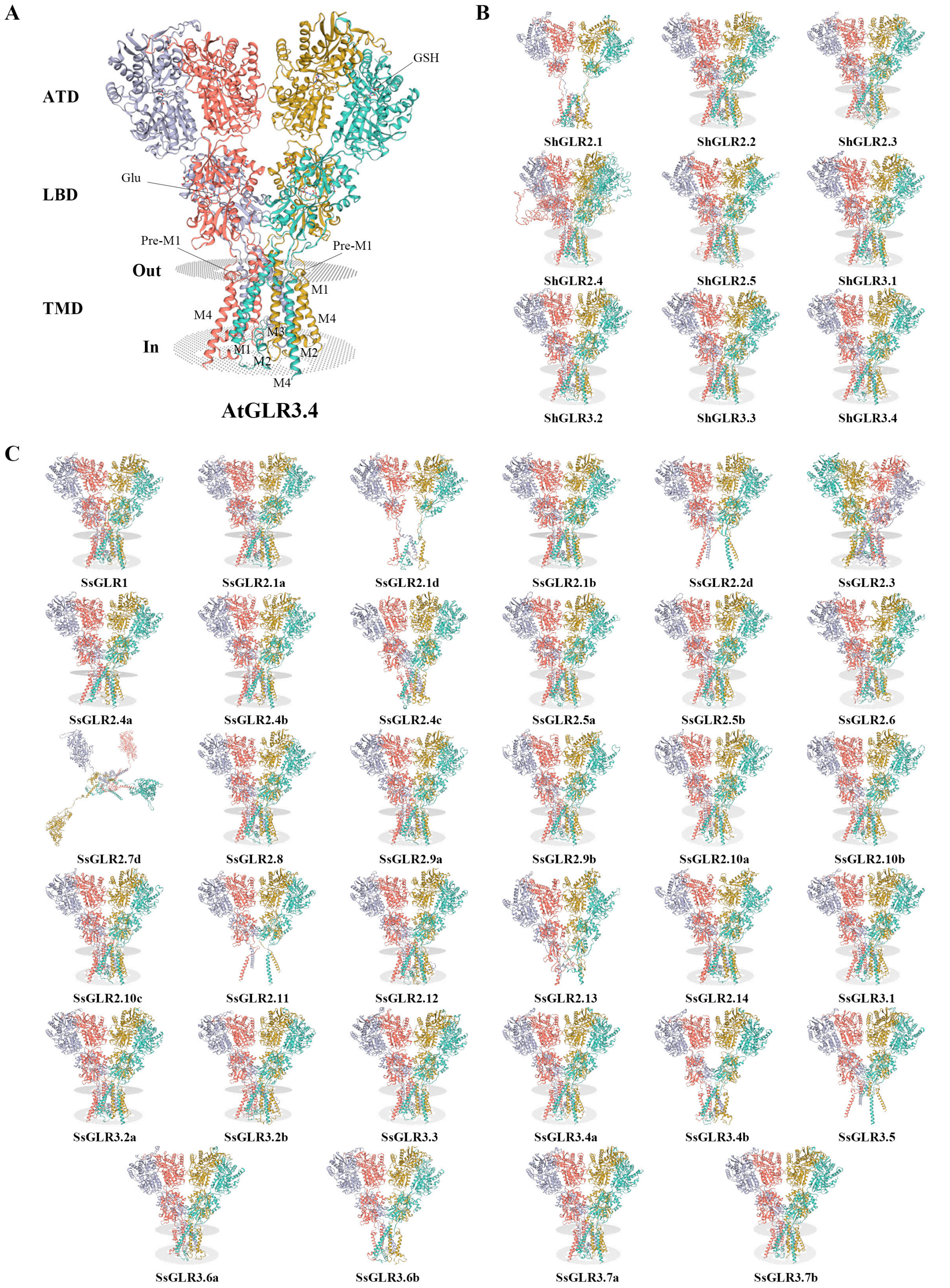

Figure S3. Prediction of tertiary structure. **(A)** The tertiary structure of AtGLR3.4. The structure label included amino-terminal domain (ATD), ligand-binding domain (LBD), transmembrane domain (TMD), glutathione molecule (GSH), glutamate molecule (Glu), beginning of M1 domain (Pr -M1), four transmembrane domains (M1, M2, M3 and M4). **(B)** The tertiary structure of ShGLRs. **(C)** The tertiary structure of SsGLRs. The structural colour scheme adopted Chian, which was divided into four subunits of different colors. Glu and GSH ligand domains were displayed in rod structure. The dotted gray strip was the predicted position of the membrane.

1 ***Saccharum* hybrid cultivar R570**

>ShGLR2.1

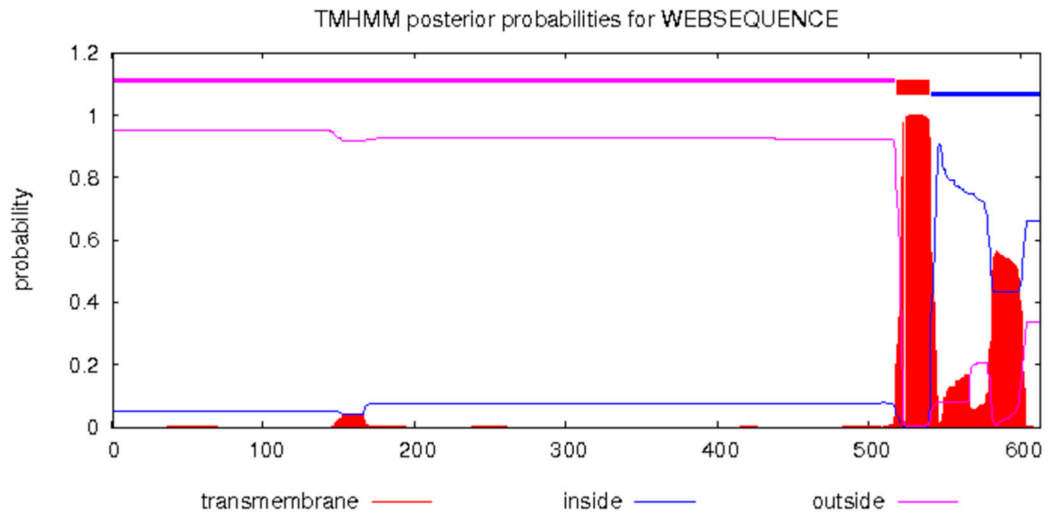

>ShGLR2.2

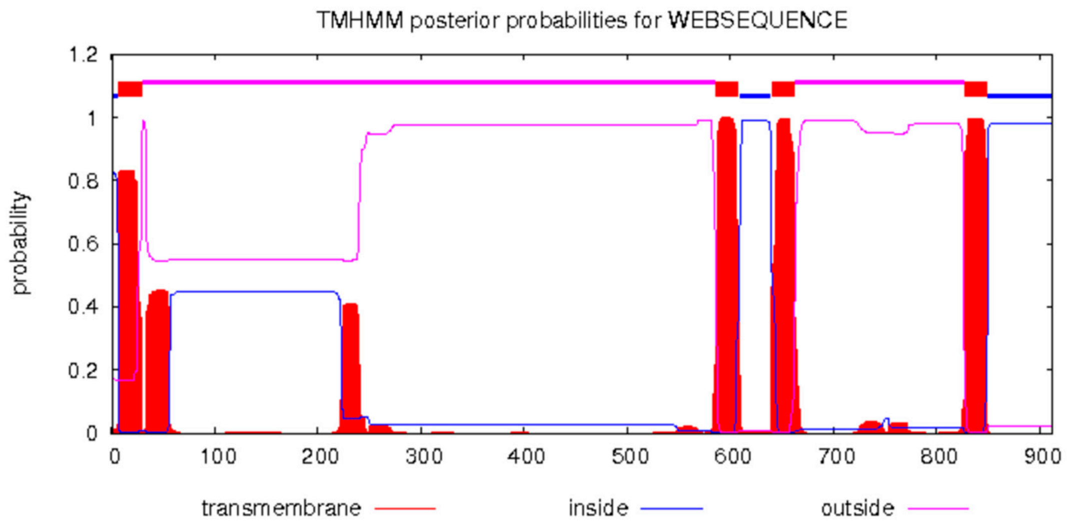

>ShGLR2.3

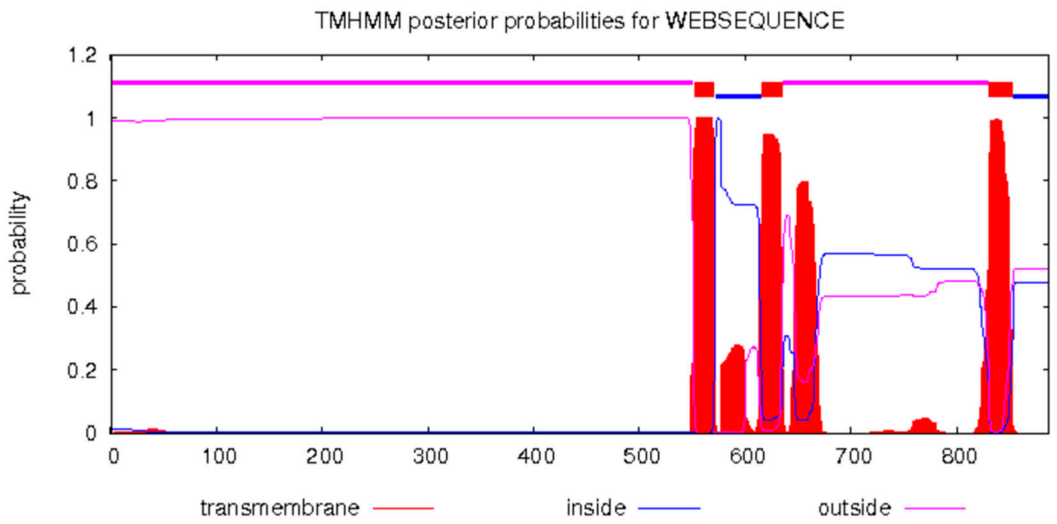

>ShGLR2.4

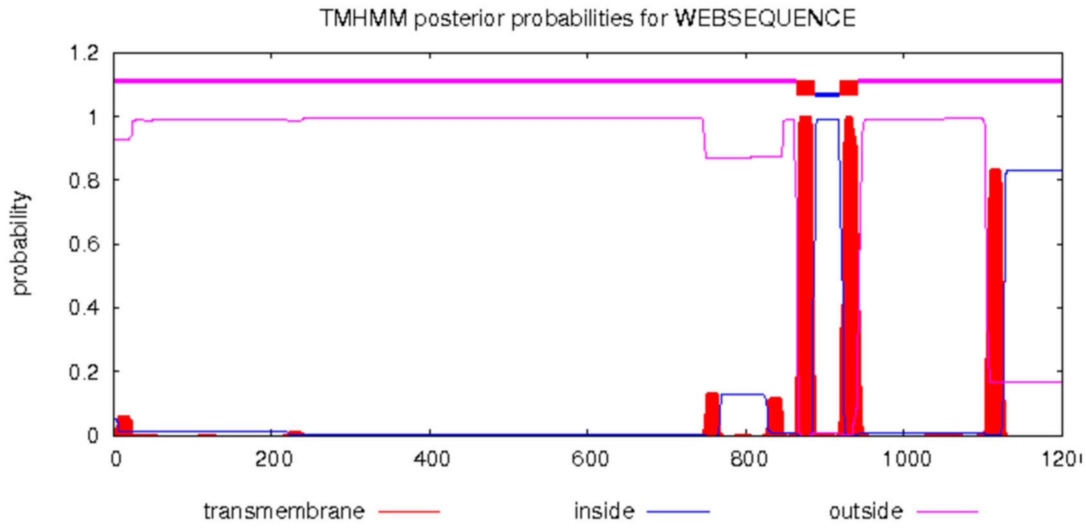

>ShGLR2.5

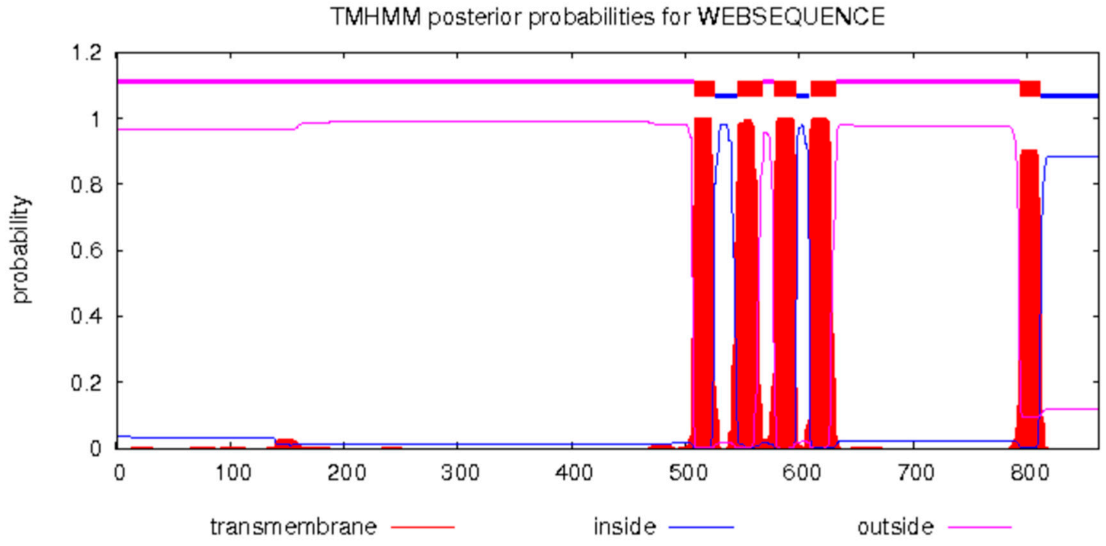

>ShGLR3.1

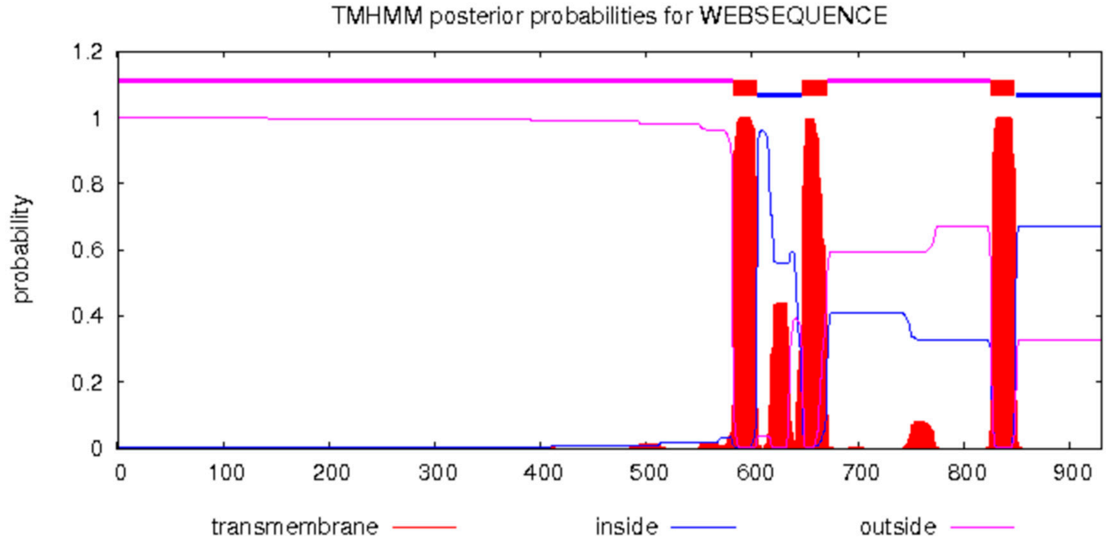

>ShGLR3.2

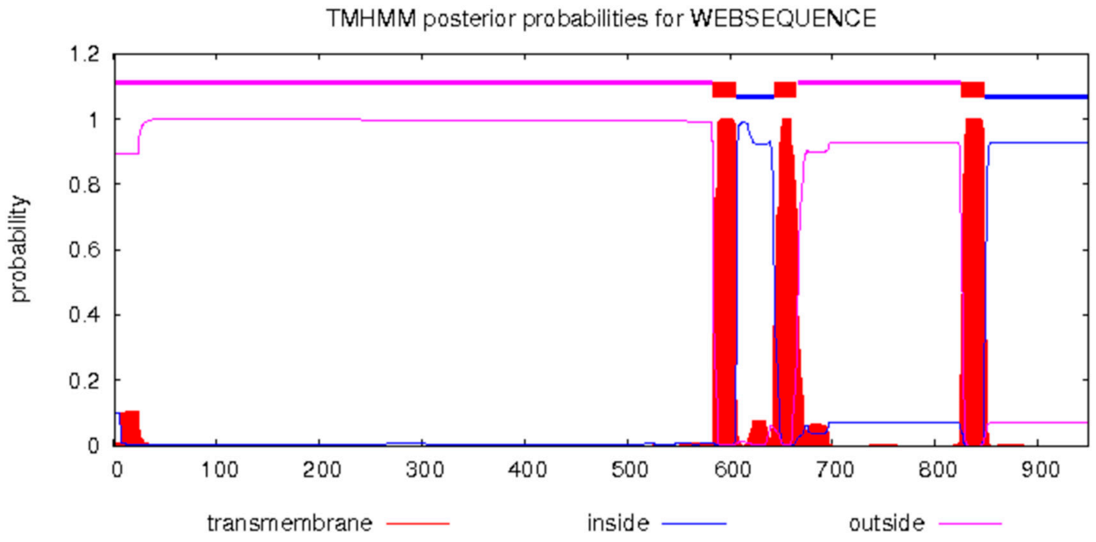

>ShGLR3.3

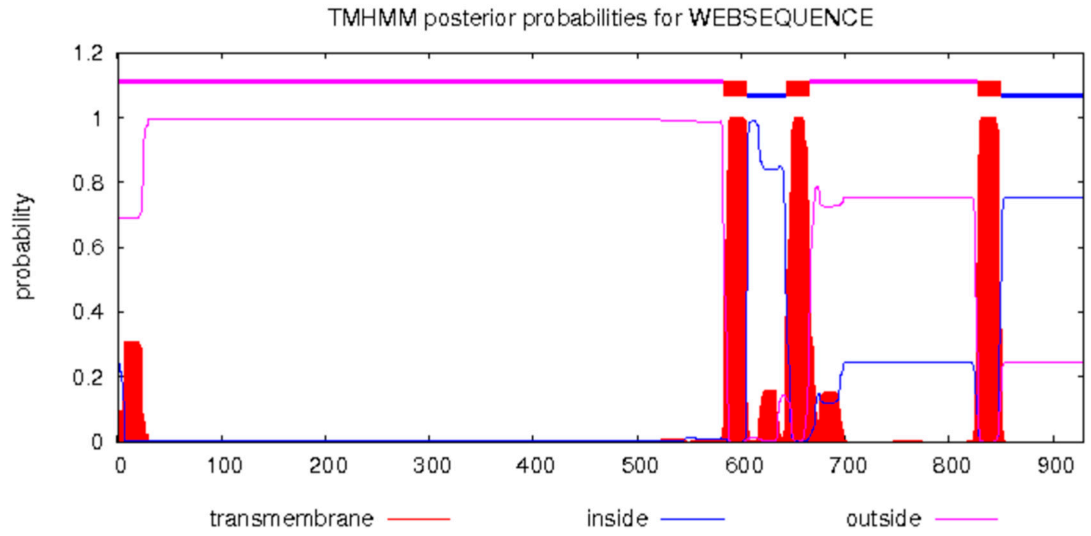

>ShGLR3.4

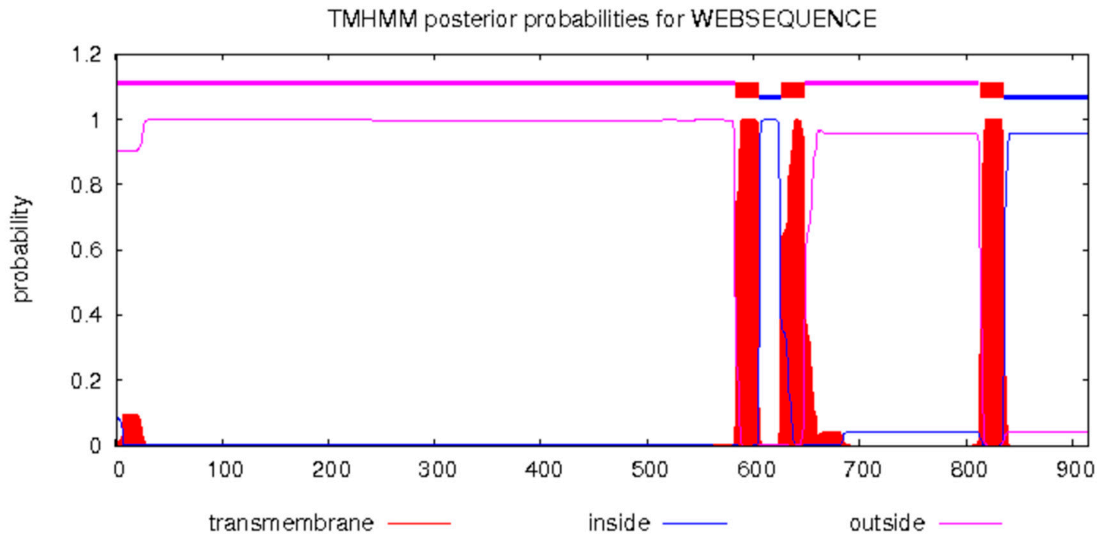

2 ***Saccharum spontaneum***

>SsGLR1

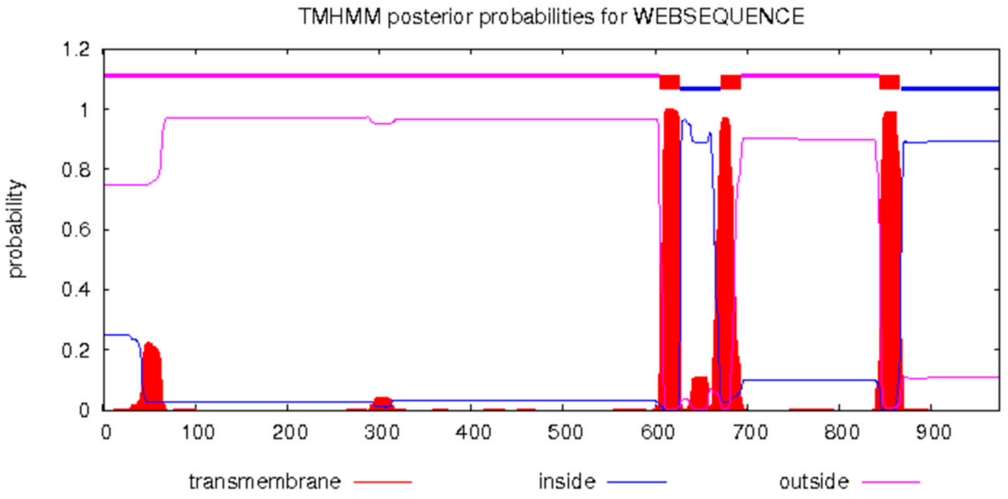

>SsGLR2.1a

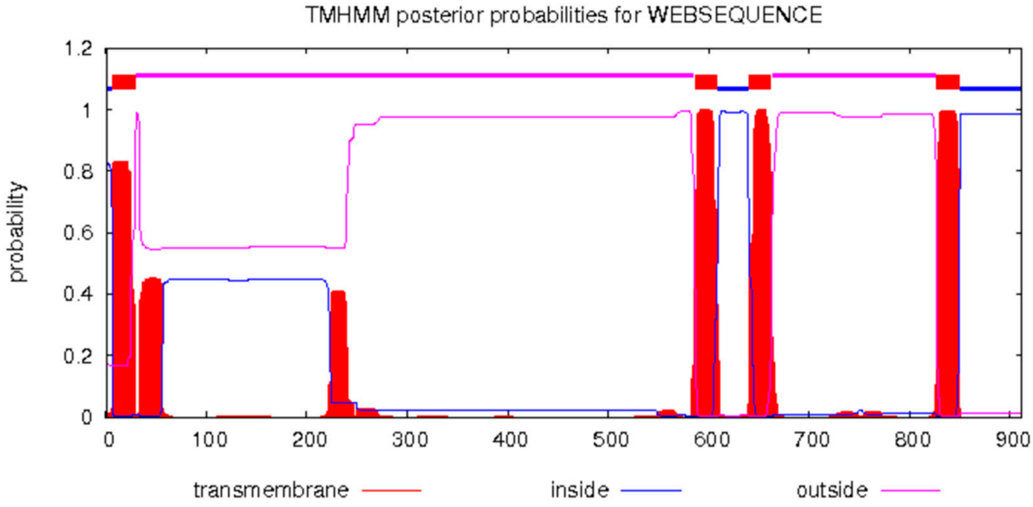

>SsGLR2.1d

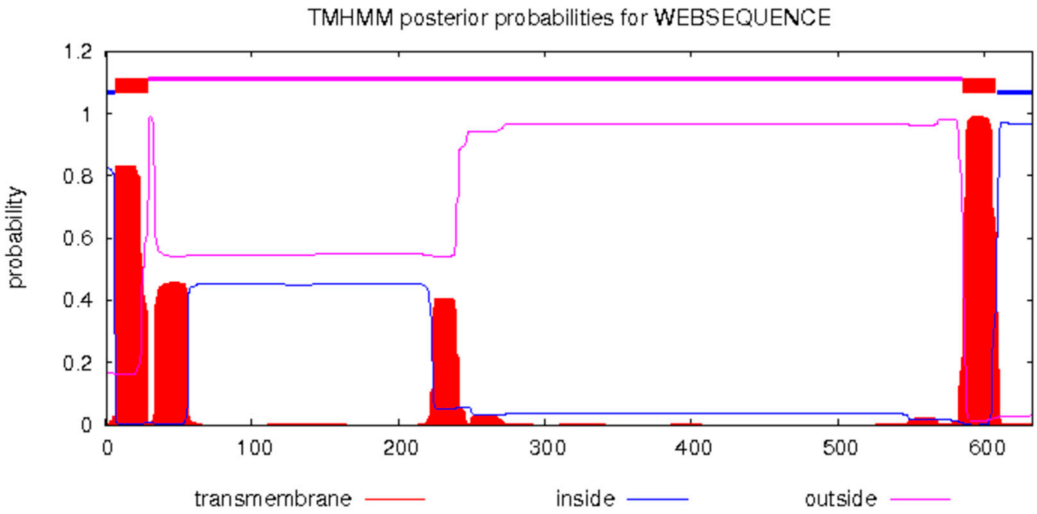

>SsGLR2.1b

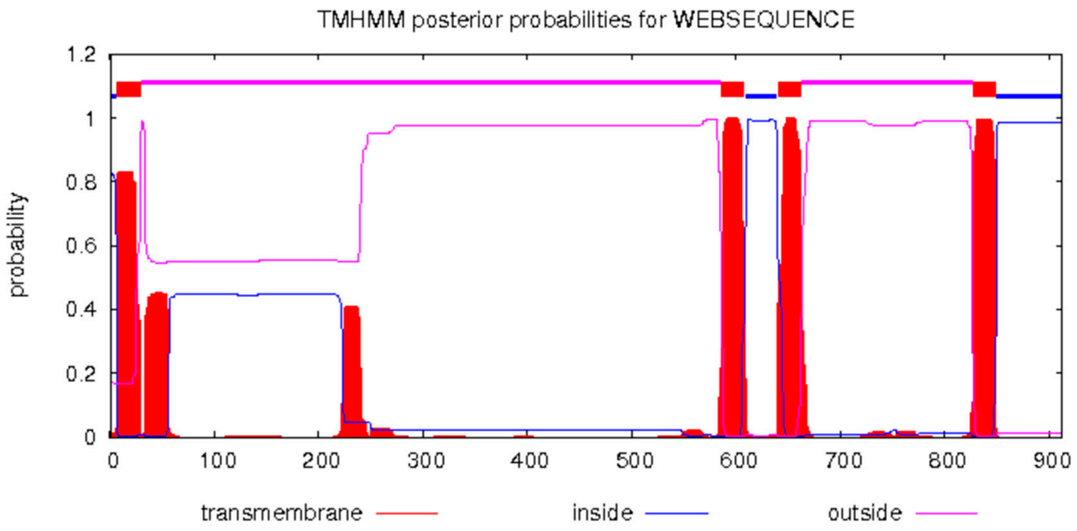

>SsGLR2.2d

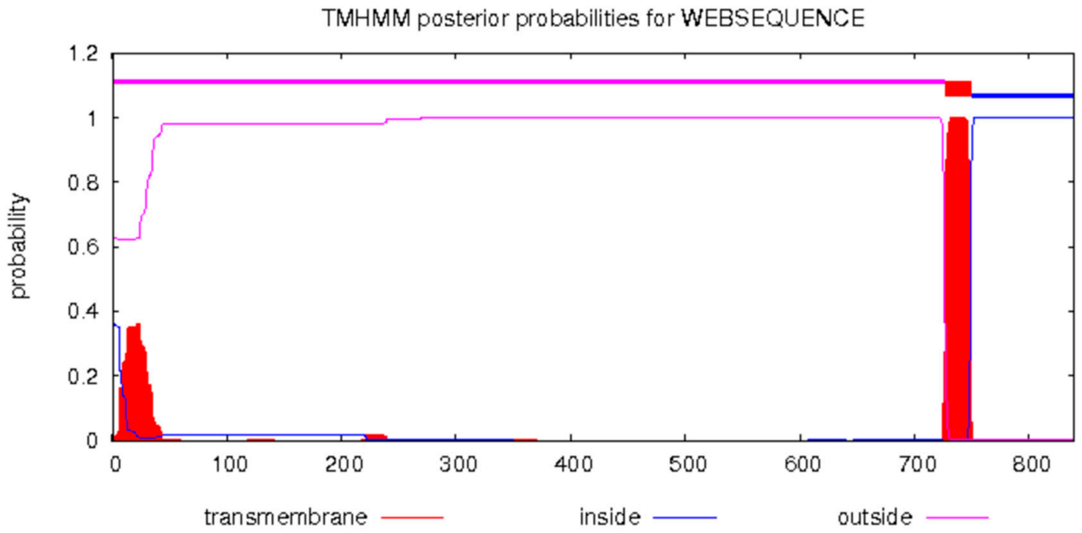

>SsGLR2.3

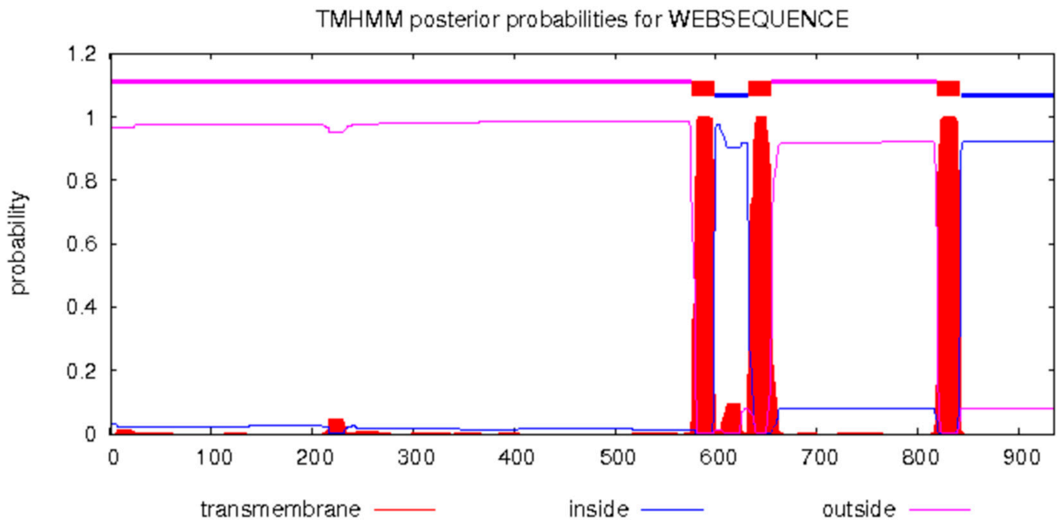

>SsGLR2.4a

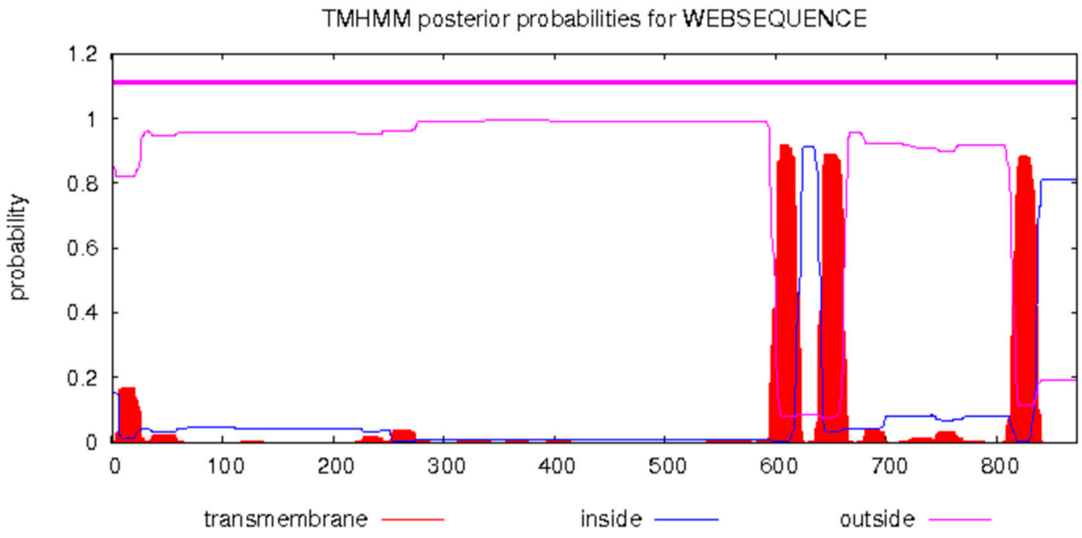

>SsGLR2.4b

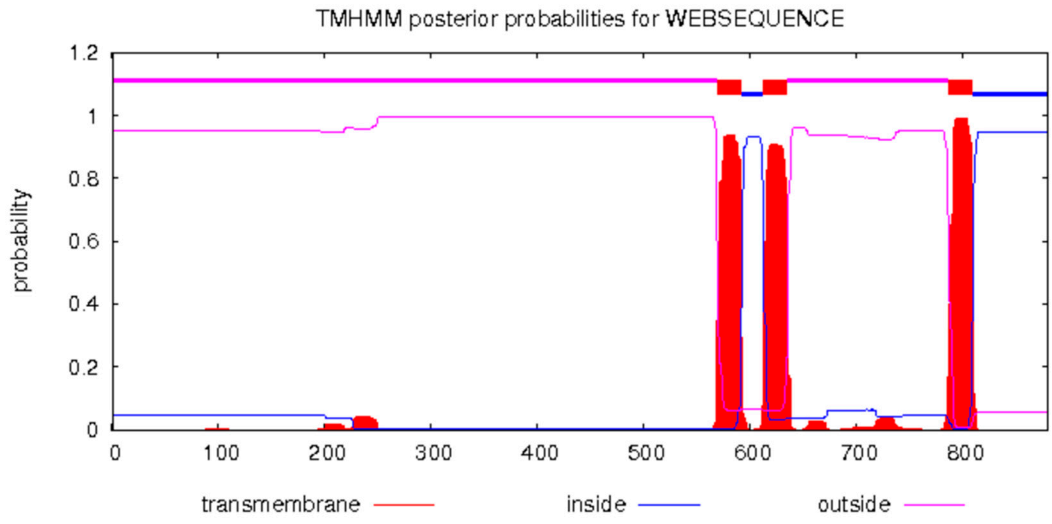

>SsGLR2.4c

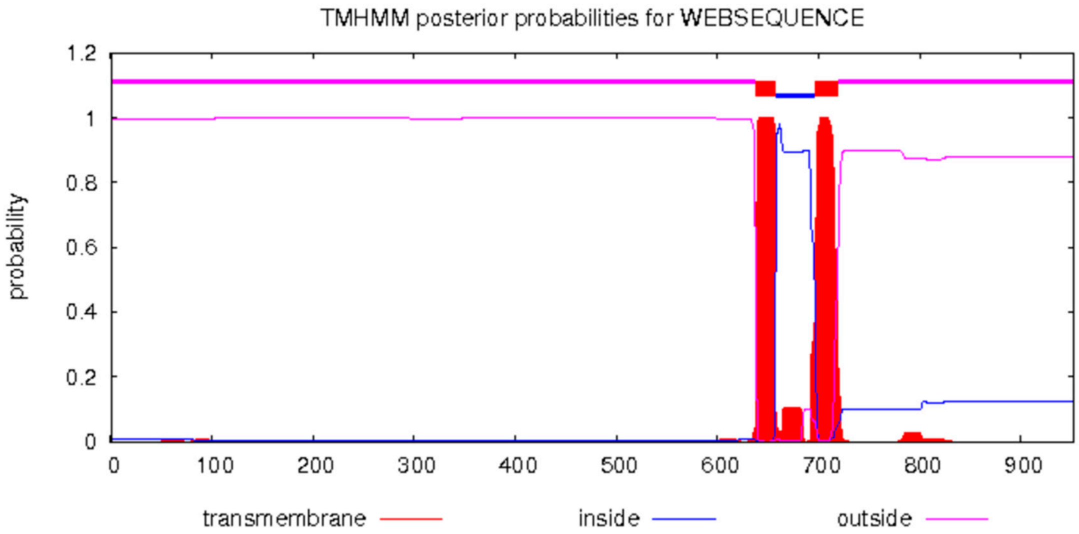

>SsGLR2.5a

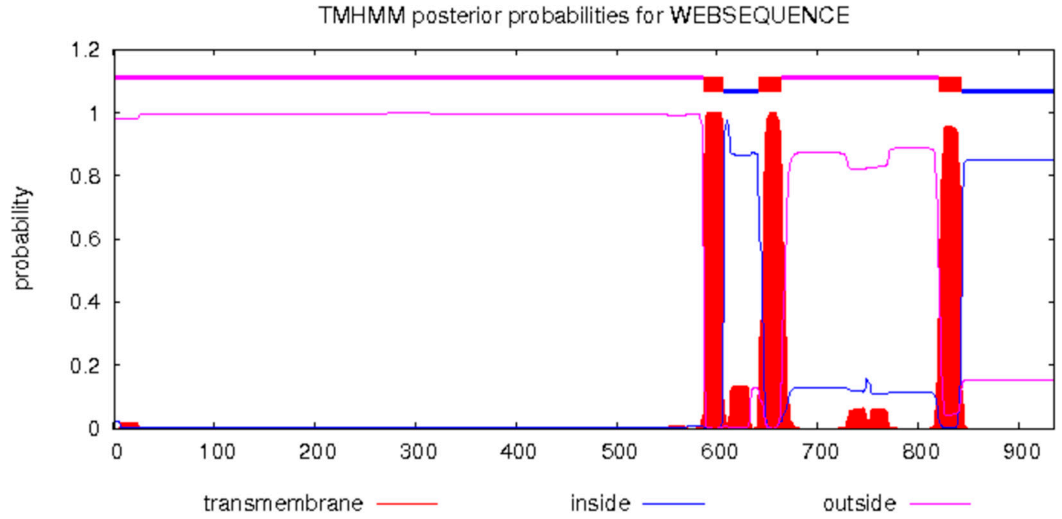

>SsGLR2.5b

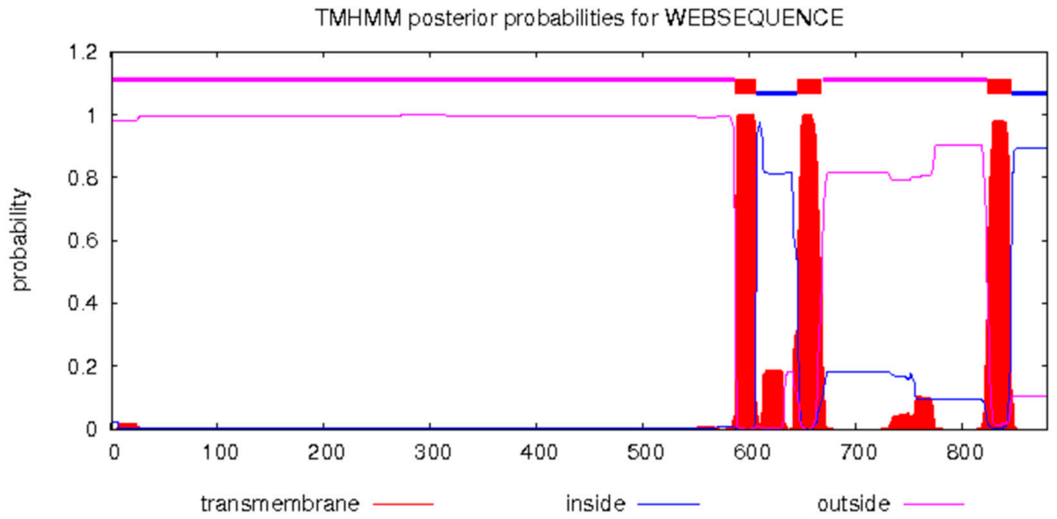

>SsGLR2.6

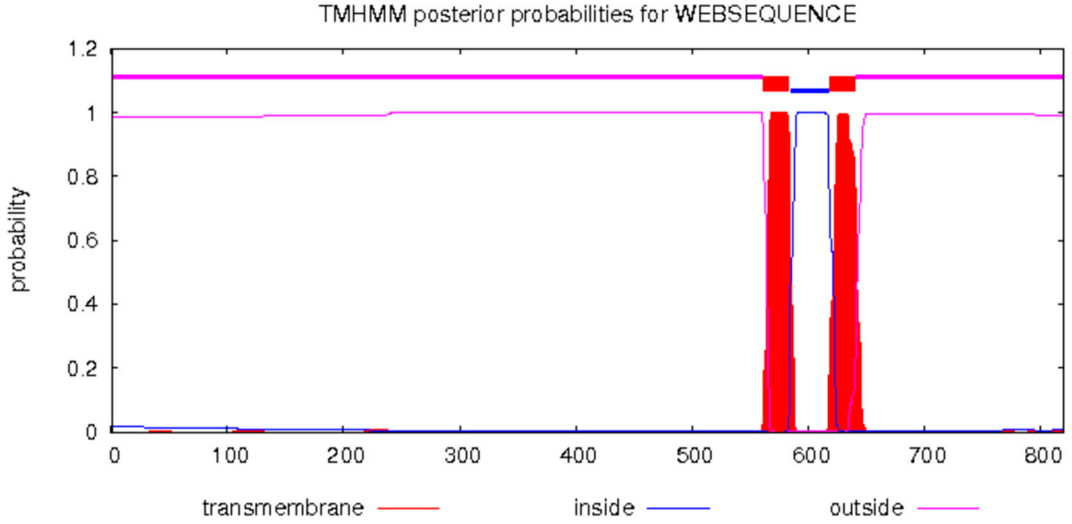

>SsGLR2.7d

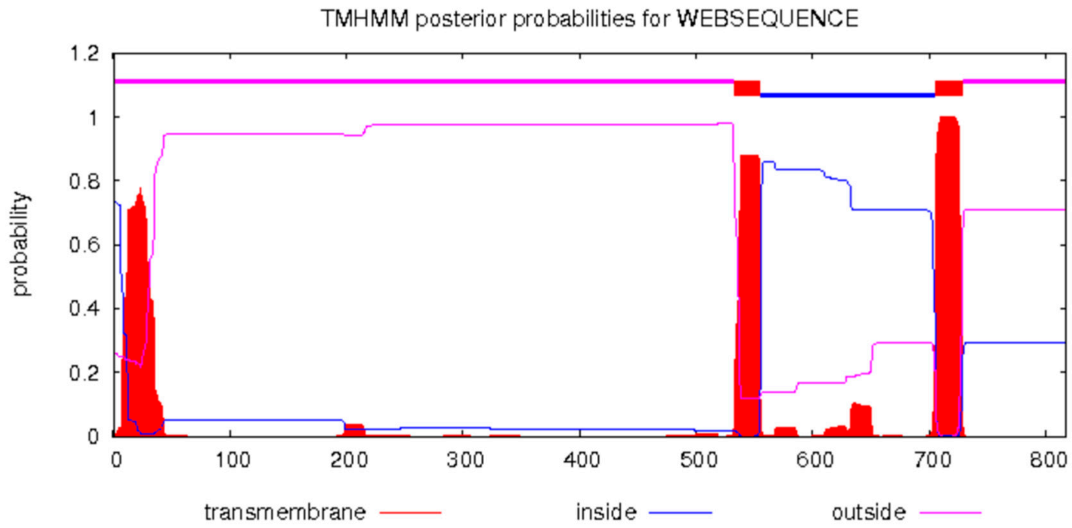

>SsGLR2.8

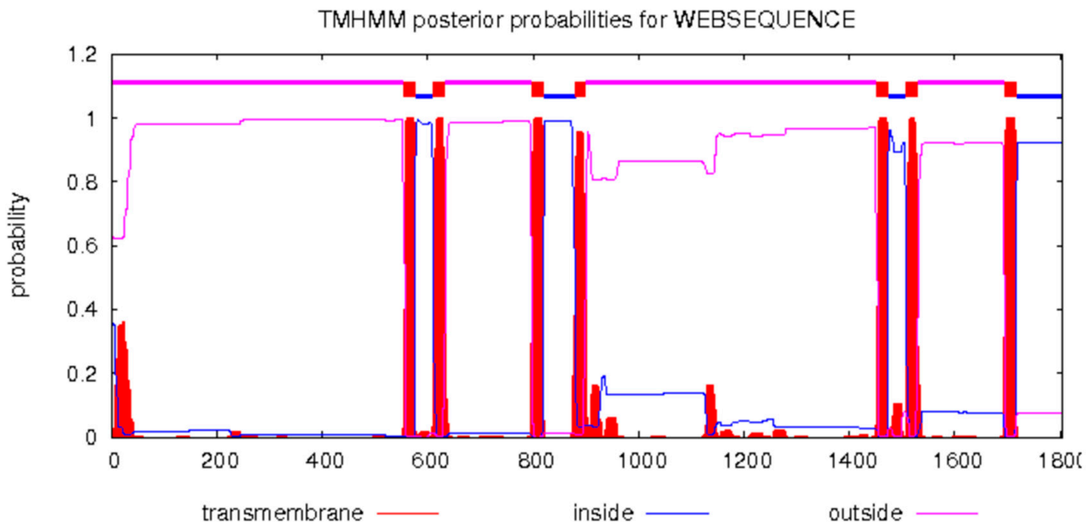

>SsGLR2.9a

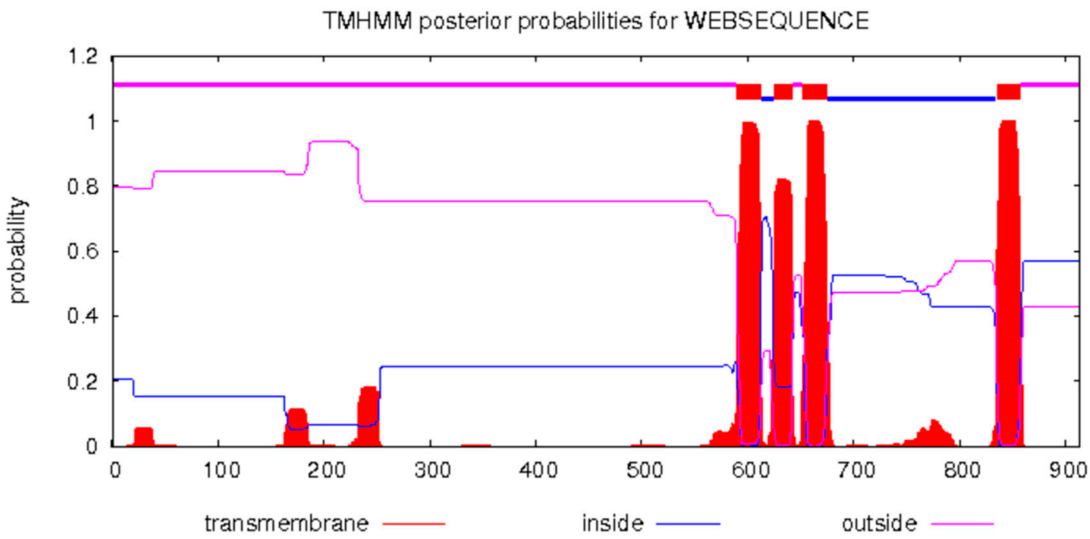

>SsGLR2.9b

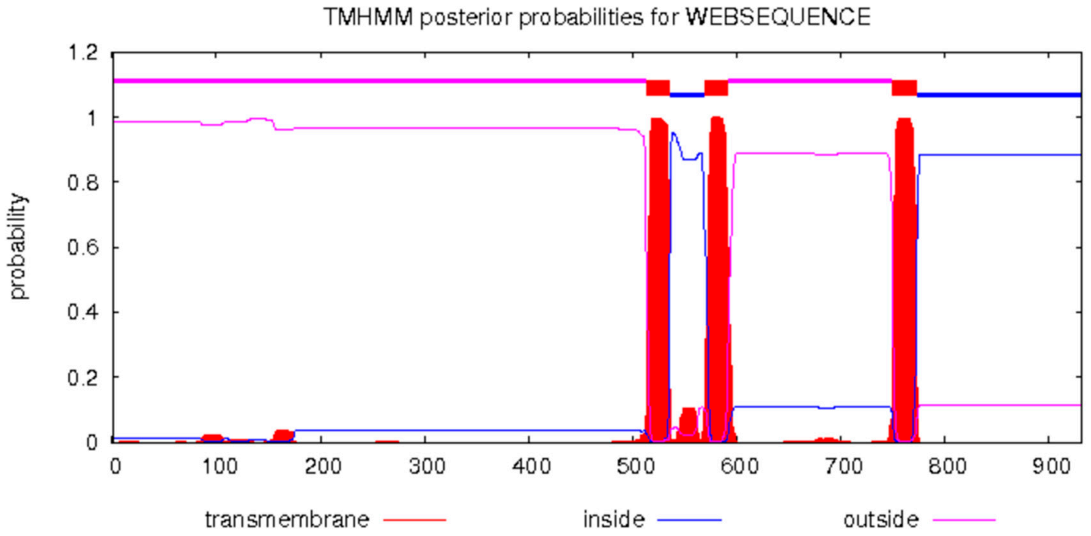

>SsGLR2.10a

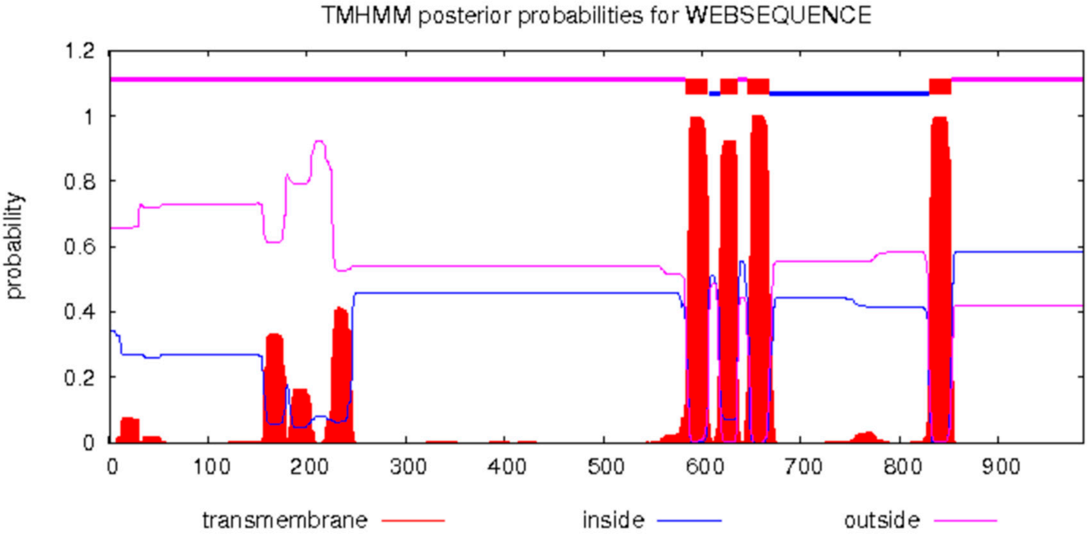

>SsGLR2.10b

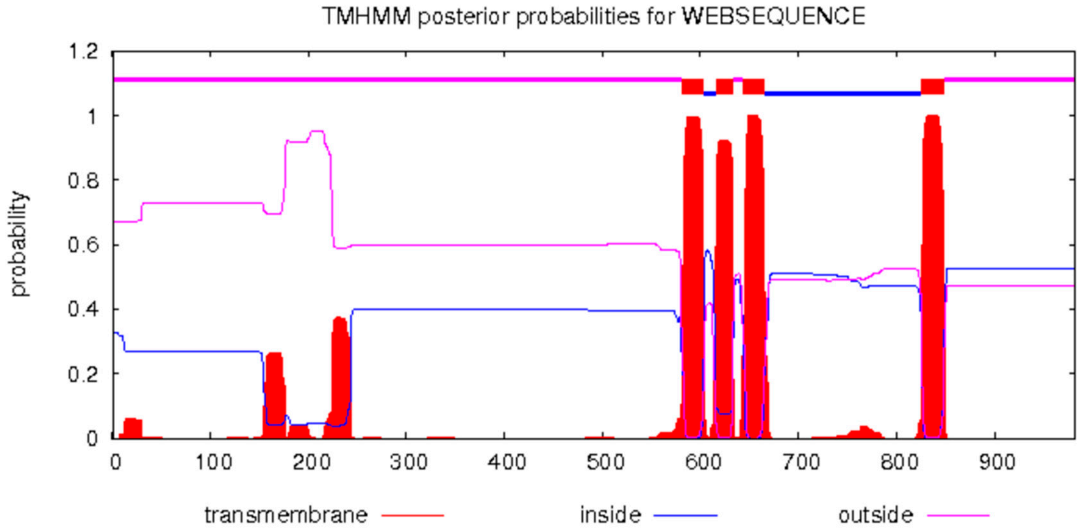

>SsGLR2.10c

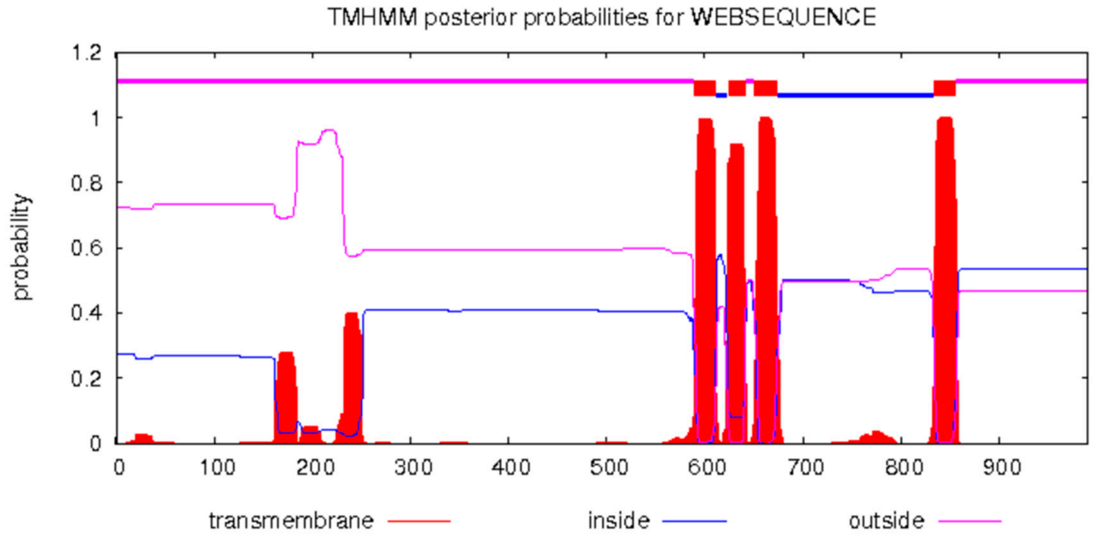

>SsGLR2.11

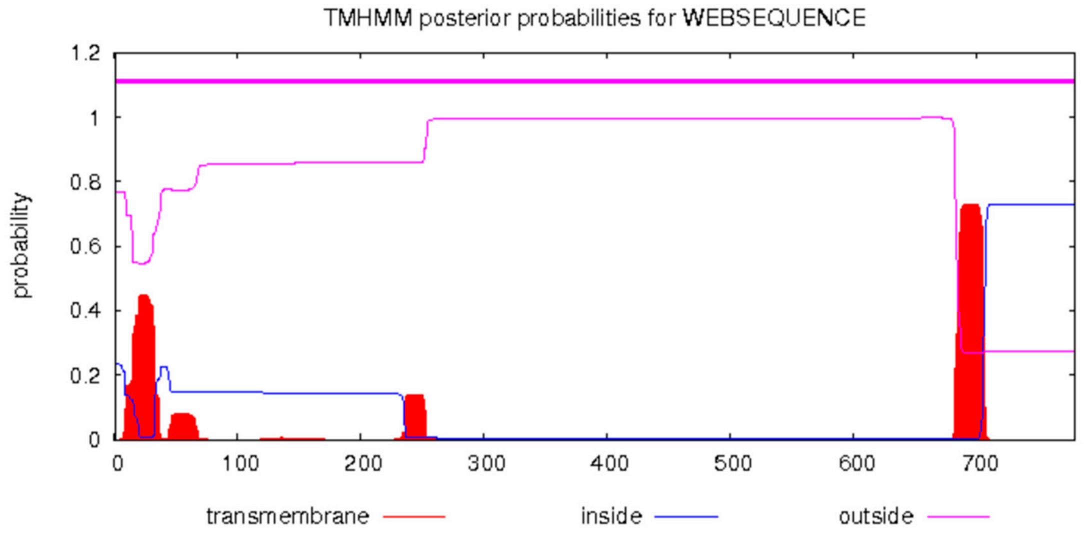

>SsGLR2.12

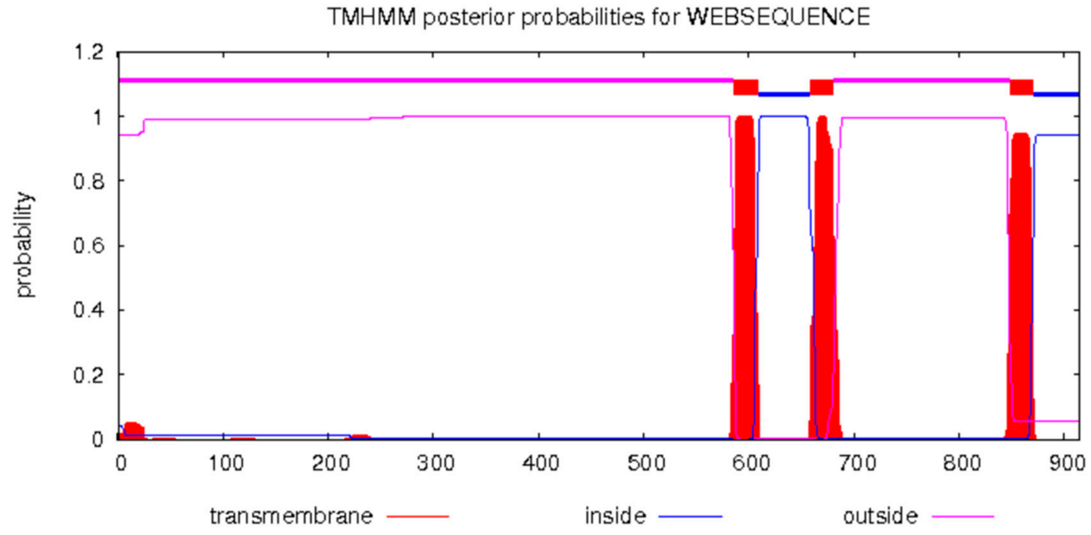

>SsGLR2.13

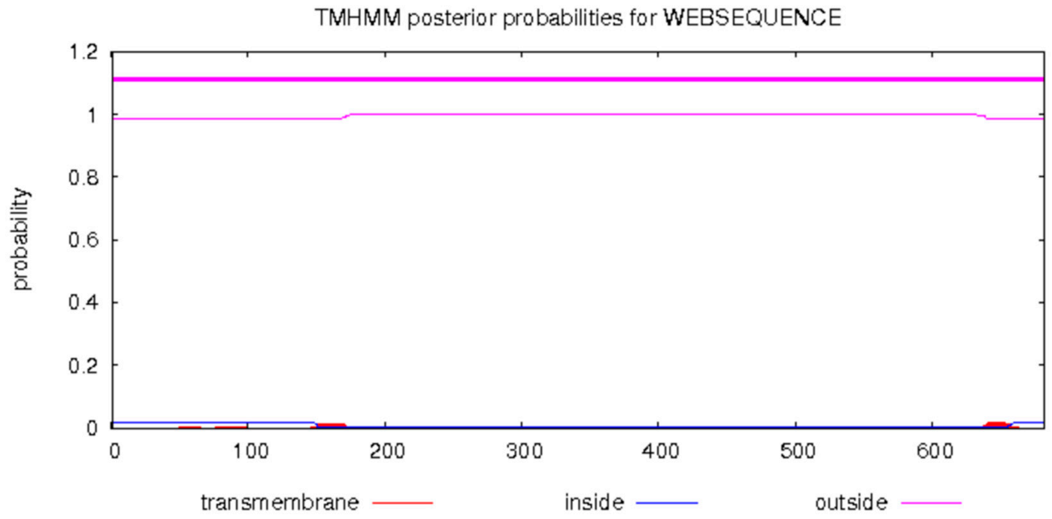

>SsGLR2.14

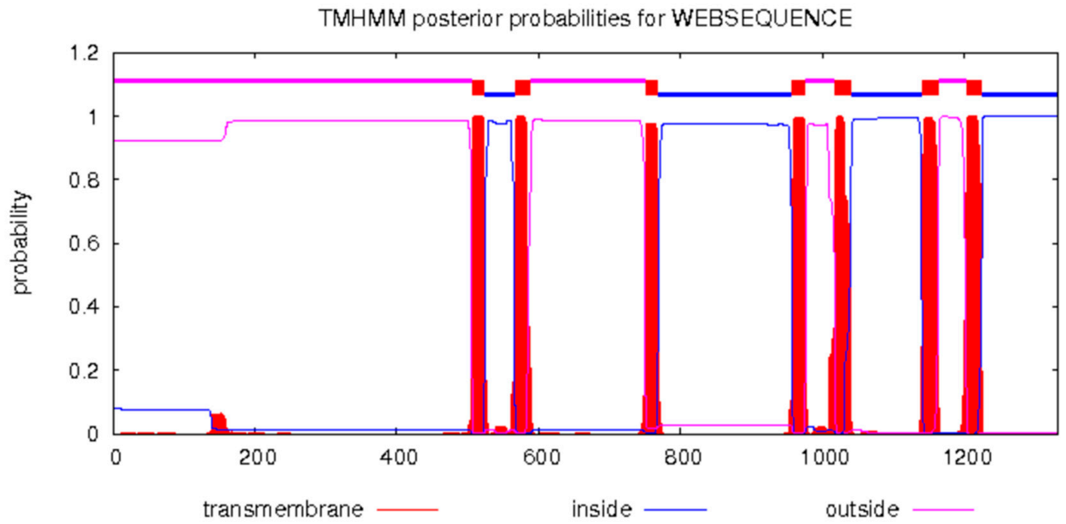

>SsGLR3.1

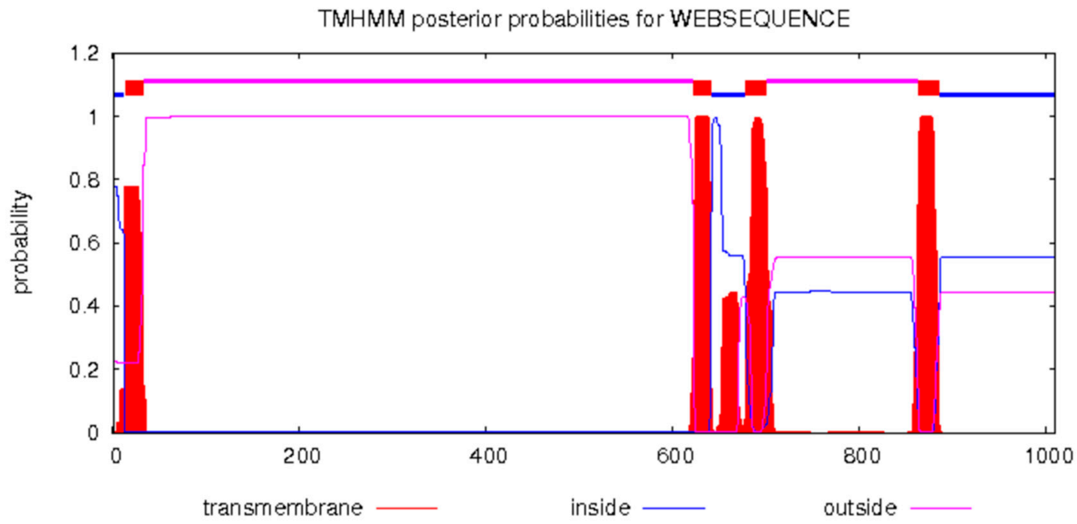

>SsGLR3.2a

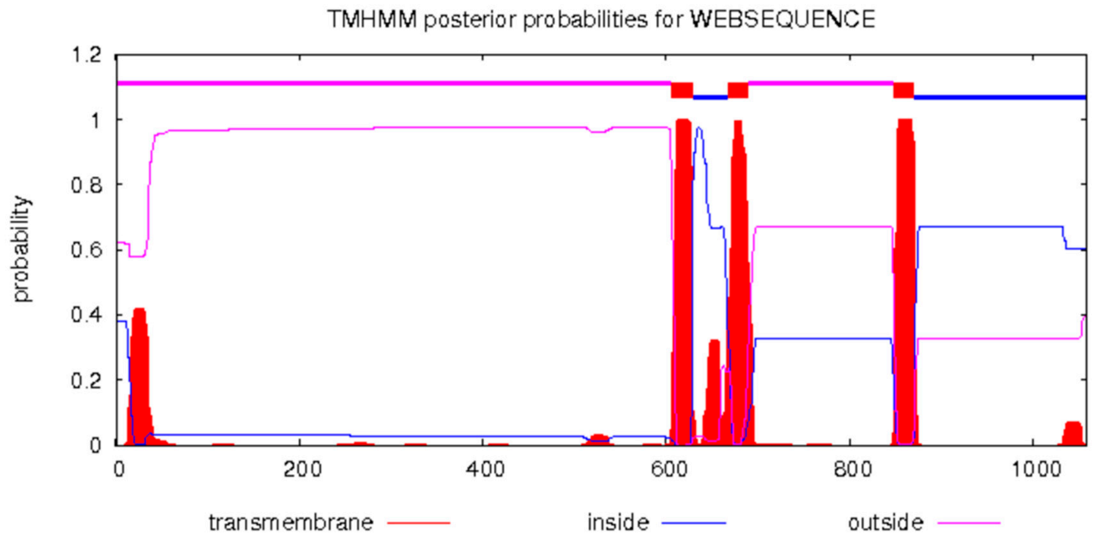

>SsGLR3.2b

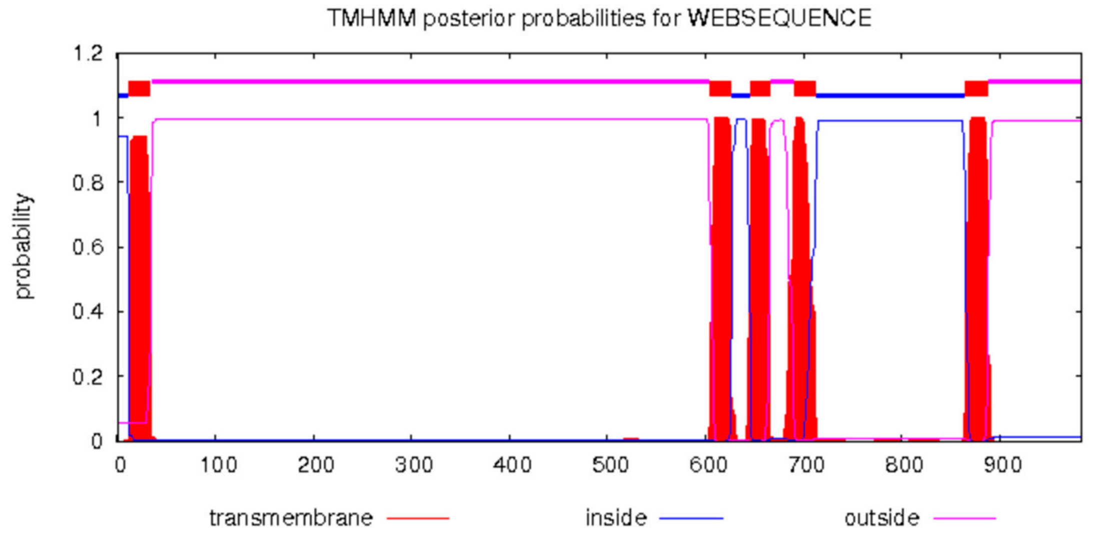

>SsGLR3.3

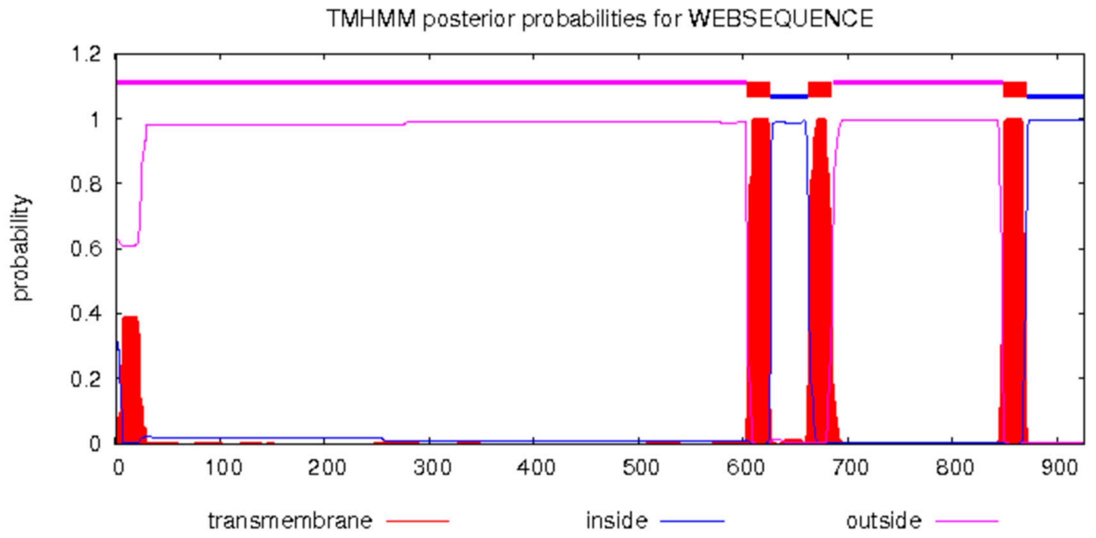

>SsGLR3.4a

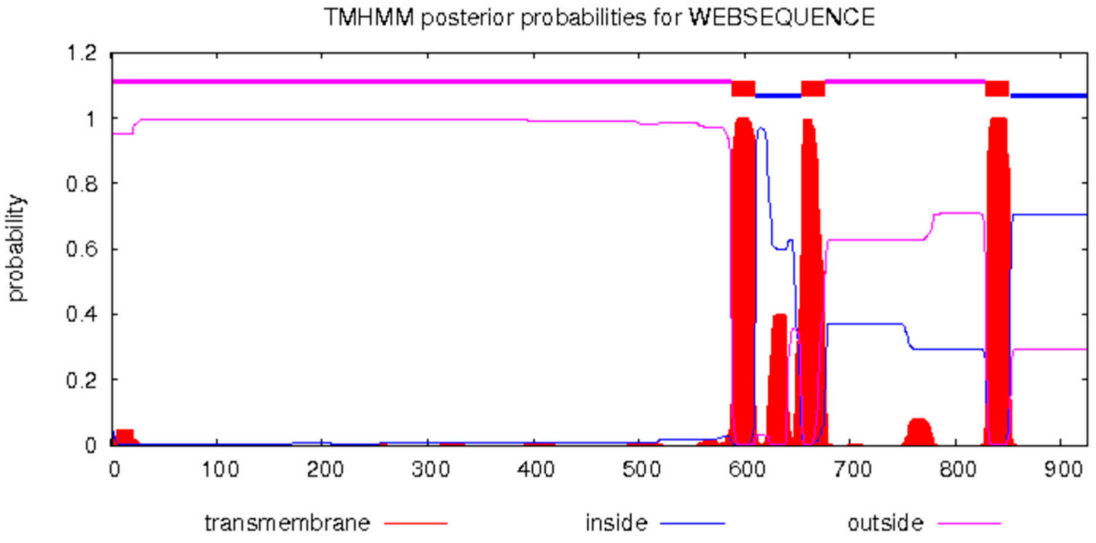

>SsGLR3.4b

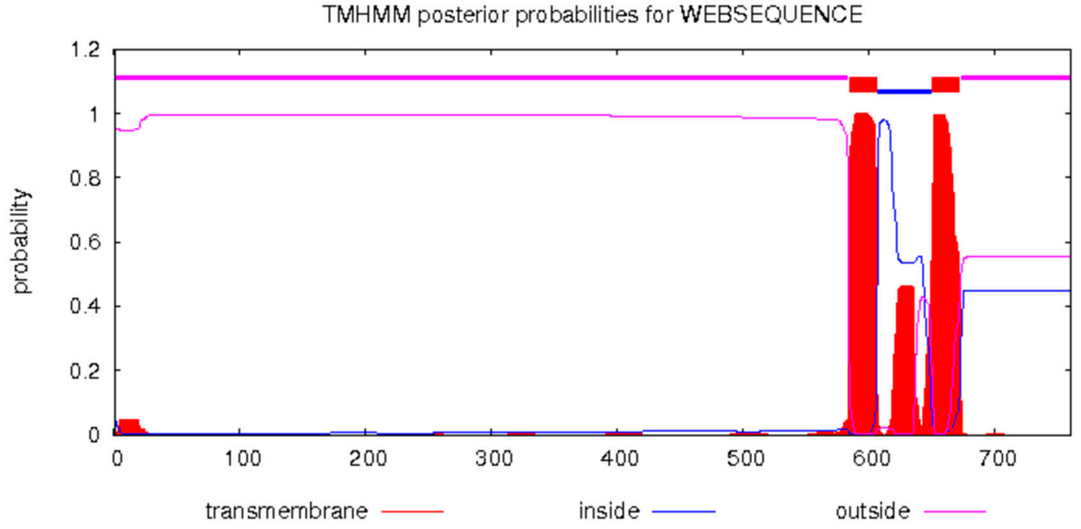

>SsGLR3.5

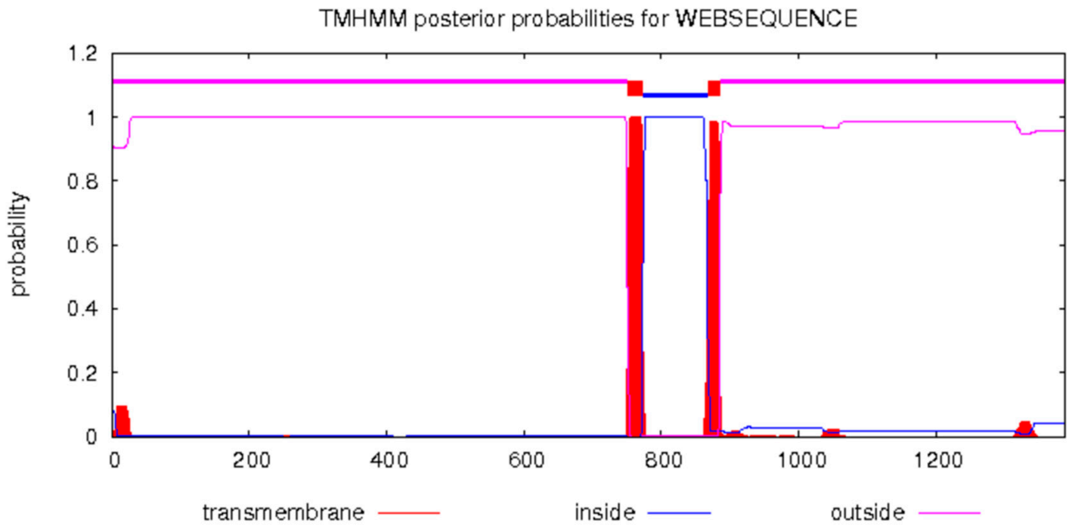

>SsGLR3.6a

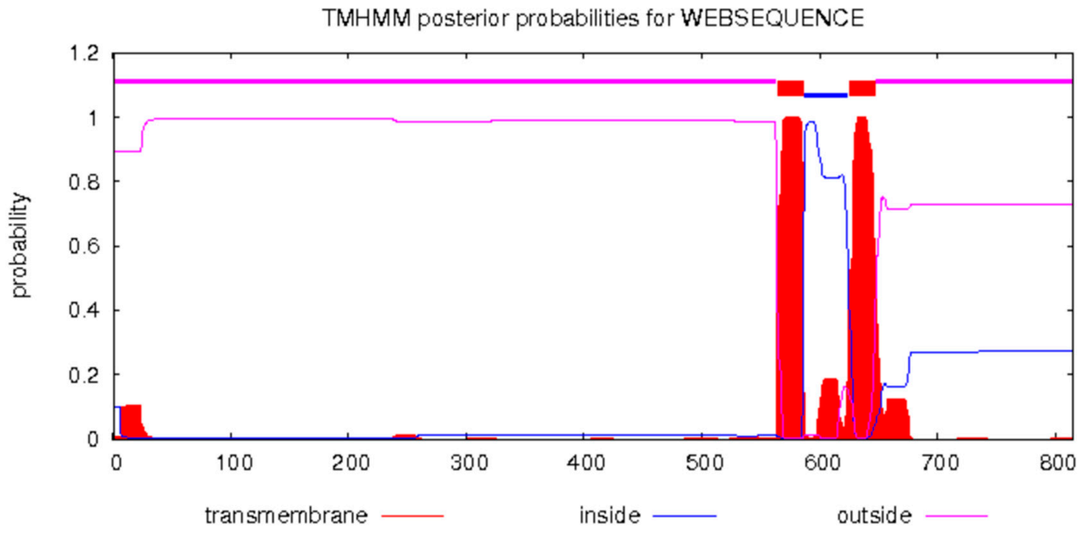

>SsGLR3.6b

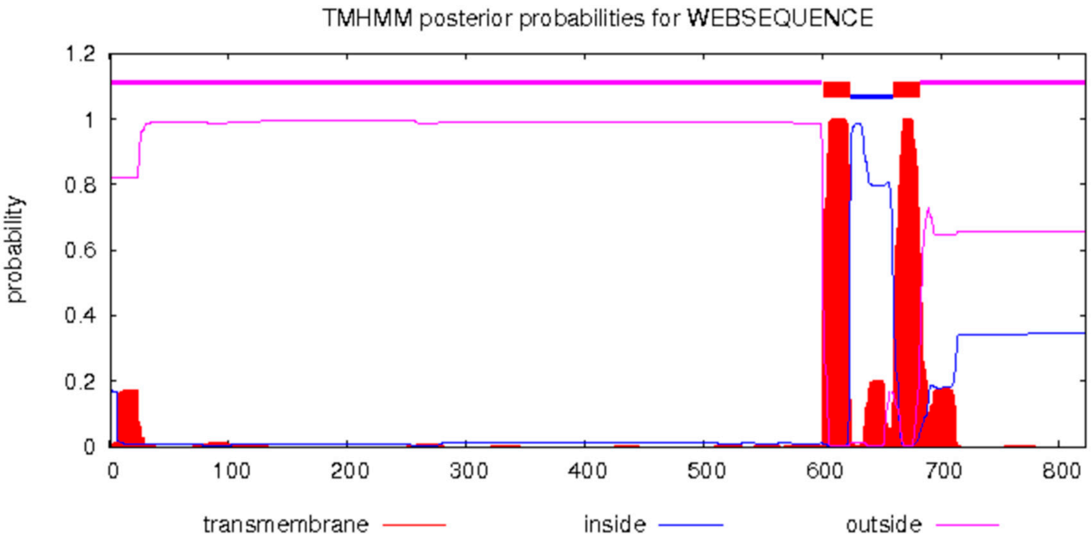

>SsGLR3.7a

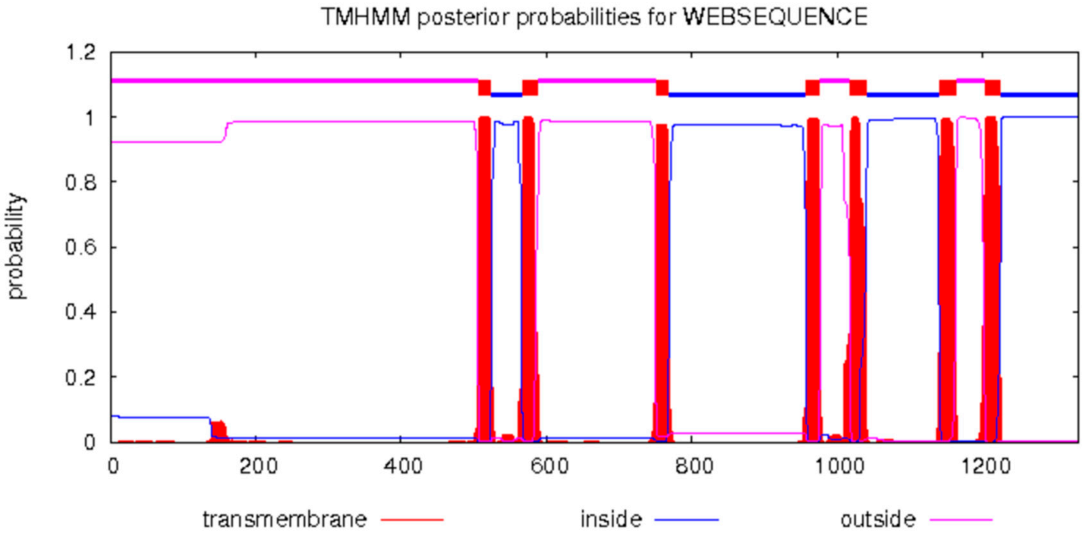

>SsGLR3.7b

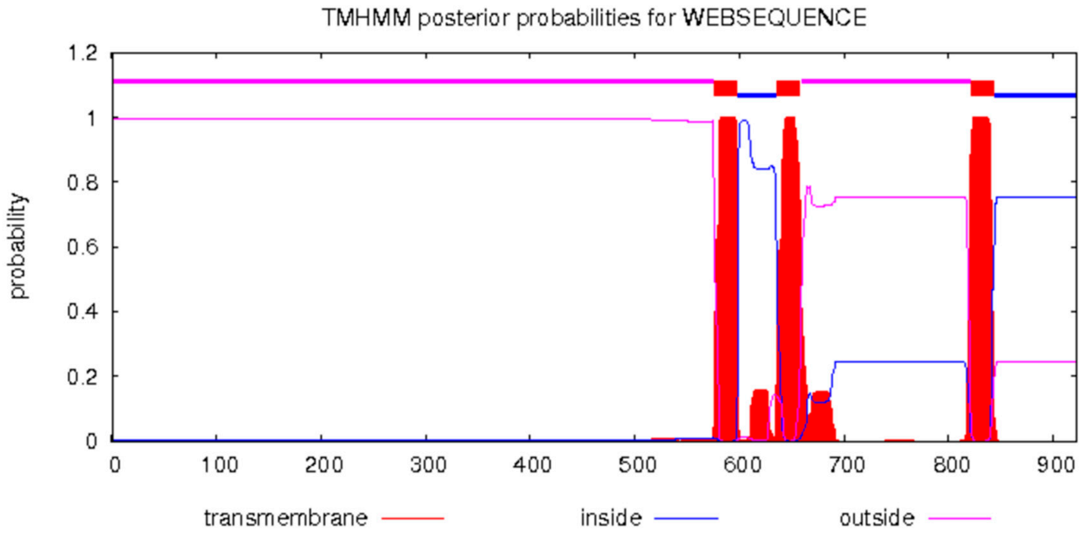

Figure S4. The transmembrane domains of SsGLRs and ShGLRs predicted by TMHMM. Inside represented the intracellular region, and the higher the value, the greater the probability that the amino acid was located in the intracellular region. Outside represented the extracellular region, and the larger the value, the greater the probability that the amino acid was located in the extracellular region. Transmembrane represented the transmembrane region, and the larger the value, indicating that the amino acid was more likely in the transmembrane region.
